# All together now: A mixed-planting experiment reveals adaptive drought tolerance in seedlings of 10 *Eucalyptus* species

**DOI:** 10.1093/plphys/kiae632

**Published:** 2024-11-29

**Authors:** Chris J Blackman, Ben Halliwell, Tim J Brodribb

**Affiliations:** ARC Centre of Excellence for Plant Success in Nature and Agriculture, School of Natural Sciences, University of Tasmania, Hobart 7001, Tasmania, Australia; ARC Centre of Excellence for Plant Success in Nature and Agriculture, School of Natural Sciences, University of Tasmania, Hobart 7001, Tasmania, Australia; ARC Centre of Excellence for Plant Success in Nature and Agriculture, School of Natural Sciences, University of Tasmania, Hobart 7001, Tasmania, Australia

## Abstract

The negative impacts of drought on plant productivity and survival in natural and crop systems are increasing with global heating, yet our capacity to identify species capable of surviving drought remains limited. Here, we tested the use of a mixed-planting approach for assessing differences in seedling drought tolerance. To homogenize dehydration rates, we grew seedlings of 10 species of *Eucalyptus* together in trays where roots of all individuals were overlapping in a common loam soil. These seedling combinations were dried down under cool and warm temperature conditions, and seedling responses were quantified from measurements of chlorophyll fluorescence (*Fv/Fm*). The day of drought (*T*) associated with an 88% decline in *Fv/Fm* (*TF*_88_) varied significantly among species and was unrelated to seedling size. No significant differences in water potentials were detected among seedlings dehydrated under warm conditions prior to leaf wilt. The rank-order of species *TF*_88_ was consistent under both temperature treatments. Under cool conditions, seedling *TF*_88_ increased with decreasing cavitation vulnerability measured on adult foliage. Under both treatments, a quadratic function best fit the relationship between seedling *TF*_88_ and sampling site mean annual precipitation. These results provide evidence for adaptive selection of seedling drought tolerance. Our findings highlight the use of mixed-planting experiments for comparing seedling drought tolerance with applications for improving plant breeding and conservation outcomes.

## Teaser

Growing seedlings of different species together overcome the size-bias problem and reveal significant and adaptive differences in the drought tolerance of different eucalyptus species.

## Introduction

Drought negatively impacts plant productivity and survivorship in natural, forestry, and agricultural systems. Drought intensity is also increasing with global heating as temperature-driven increases in vapor pressure deficit rapidly draws water out of plants and dehydrates surrounding soils (Grossiord et al. 2020; Vicente-Serrano et al. 2022). Given this process is leading to increased tree mortality in forest systems (Allen et al. 2015; Hammond et al. 2022) and causing catastrophic crop losses during flash drought events (Christian et al. 2021), there is an urgent need to assess species' drought mortality risk and identify crop species and/or varieties capable of surviving water deficit. However, achieving these goals is challenging due to the complexity of plant physiological responses to drought and limited capacity to characterize drought tolerance across large numbers of species and/or genotypes.

Due to their relatively small size and experimental tractability, seedlings are ideally suited for characterizing drought tolerance in plants. In evergreen seedlings, there are 2 pathways to surviving a lack of rainfall; one is to conserve water, while the other is to build more resilient leaves, stems, and roots that can tolerate increasing water deficit for longer periods of time. In natural systems where seedlings are in close competition for both light and water, sacrificing growth in favor of conserving water during drought is unlikely to be selected because water conserved in the soil by a frugal individual will be used by another less frugal neighbor (Markesteijn and Poorter 2009). Hence, it is variation in the seedling's capacity to tolerate water deficit that is likely to be under selection and of interest for breeding and restoration. While evidence of strong selection for increased drought tolerance has been reported in seedlings distributed across environmental gradients in water availability (Engelbrecht et al. 2007; Baltzer et al. 2008), pot experiments cannot easily compare drought tolerance due to the inseparable link between water use, which is strongly influenced by plant size, and the rate of dehydration of an individual in a pot (Juenger and Verslues 2023). This limitation applies equally to phenotyping approaches in model plants such as Arabidopsis (*Arabidopsis thaliana*), where “drought tolerance” is commonly conflated with any change in conditions that decreases plant growth rate. To address this issue, researchers either account for differences in plant size in their statistical models (e.g. Ammitzboll et al. 2020) or try to standardize rates of soil dehydration among experimental plants by tracking changes in pot mass or soil water potential and adding water back to individuals with higher rates of water use (e.g. Duan et al. 2014, 2018). However, these approaches are often arduous and time consuming or involve complex irrigation systems linked to pots on balances, which limits capacity for measuring drought tolerance across large numbers of species and/or genotypes—although high-throughput autonomous and robotic systems are rapidly advancing (Atefi et al. 2021).

One relatively straight forward approach for comparing seedling drought tolerance is to use a common garden type experiment with seedlings or saplings grown together in pots or trays with access to the same limited soil volume (Ranney et al. 1991; Izanloo et al. 2008; Candido-Ribeiro and Aitken 2024). This mixed-planting approach is assumed to homogenize rates of dehydration among individuals during drought. However, the extent to which this occurs will likely depend on whether the roots of each seedling overlap with those of its neighbors and remain in contact with the soil during drought. Using fine-textured soils with small pore sizes and hence high hydraulic integrity will also help ensure plants remain well connected with each other through the soil (Sperry et al. 1998; Hultine et al. 2006). If a similar rate of dehydration among plants is achieved, differences in the timing of mortality among individuals should represent differences in seedling drought tolerance, and not differences in size and water use, including residual conductance following stomatal closure. Additionally, planting seedlings together in a confined tray will nullify the effect of traits such as rooting depth, which can otherwise affect the timing of mortality in field trials where seedlings that prefer root growth are able to access deeper soil water (Poorter and Markesteijn 2008; Aspinwall et al. 2023).

In this study, we used a mixed-planting experiment to compare differences in seedling drought tolerance among 10 species of *Eucalyptus* from widely contrasting climates in Tasmania, Australia. Seedlings of each species were germinated in separate pots and then grown together in trays before being dehydrated to critical levels of drought damage. To maximize homogeneity in the rate of dehydration among individuals, we used a fine-textured loam soil and grew seedlings of each species together for 6 wk before imposing drought. During dehydration, we characterized species drought tolerance using a threshold of decline in chlorophyll fluorescence (*Fv/Fm*) linked to catastrophic xylem embolism (Brodribb et al. 2021; Fortunel et al. 2023; Tonet et al. 2023). We tested for consistency in the rank-order of species drought tolerance by growing and dehydrating seedlings under warm and cool temperature conditions, respectively. We also measured predawn water potentials in seedlings from a subset of trays under the warm treatment conditions to test for homogeneity in plant water status during drought. The time for seedlings of each species to reach critical thresholds of drought stress was compared against species cavitation vulnerability measured previously in adult leaves (Hartill et al. 2023) and the mean annual precipitation (MAP) at each species' sampling site. If similar selective pressures exist across developmental stages, we hypothesized that seedling drought tolerance across species would (i) increase with decreasing cavitation vulnerability measured in adult foliage and (ii) increase with decreasing sampling site rainfall.

## Results

At the start of each dry-down phase, seedling height varied significantly between species (delta akaike information criterion [dAIC] > 70; Table 1) but did not differ significantly between warm and cool treatments (*Anova*: χ^2^_df__=__1_ = 0.83, *P* = 0.36). Mean seedling height ranged from 15.4 cm in *Eucalyptus vernicosa* to 27.6 cm in *E. viminalis* under warm treatment conditions and from 10.4 cm in *E. vernicosa* to 28.0 cm *E. viminalis* under cool treatment conditions (Table 2). While seedlings of *E. vernicosa* and *E. viminalis* were the shortest and tallest in both treatments, respectively, the overall rank-order correlation between treatments in species seedling height at the start of each dry-down phase was not significant (*ρ* = 0.5, *P* = 0.14), indicating species-specific growth responses to the 2 temperature conditions.

**Table 1.** AIC comparison of 2 linear mixed models testing the effect of (i) treatment (fixed effect) and species identity and tray (random effects) on seedling height and (ii) treatment and seedling height (fixed effects) and species identity and tray (random effects) on seedling drought tolerance (*TF*_88_)

Model 1	AIC	Model 2	AIC
Height (Hght)		*TF*_88_	
Hght_fit_all	1,064.5	*TF*_88__fit_all	554.5
Hght_fit_SpID	**1,062**.**8**	*TF*_88__fit_SpID	**554**.**2**
Hght_fit_Tray	1,136.0	*TF*_88__fit_Tray	597.0
Hght_fit_noRand	1,134.0	*TF*_88__fit_noRand	595.0

The significance of random effects was tested via model comparison by sequentially dropping random effects and comparing the AIC of candidate models. Bold values indicate the best-fit model.

**Table 2.** List of the 10 *Eucalyptus* species in the study, including mean (±Se), seedling height (cm), and *TF*_88_ (days) recorded for each species under the warm and cool treatment conditions

Species	Species ID	Warm treatment		Cool treatment	
		Height	*TF*_88_	Height	*TF*_88_
*E. coccifera*	Euc_cocc	18.9 ± 1.0	7.6 ± 0.2	14.7 ± 0.8	15.7 ± 0.5
*E. johnstonii*	Euc_john	20.1 ± 0.7	7.1 ± 0.2	19.2 ± 1.2	14.8 ± 0.5
*E. nitida*	Euc_niti	24.2 ± 1.4	7.6 ± 0.2	22.4 ± 1.0	14.4 ± 0.4
*E. obliqua*	Euc_obli	17.0 ± 1.4	6.7 ± 0.2	25.3 ± 0.6	13.1 ± 0.6
*E. pulchella*	Euc_pulc	19.7 ± 1.7	8.0 ± 0.3	23.2 ± 1.6	14.9 ± 0.5
*E. regnans*	Euc_regn	21.6 ± 0.8	6.7 ± 0.2	21.1 ± 1.2	13.7 ± 0.4
*E. risdonii*	Euc_risd	17.9 ± 1.0	7.8 ± 0.1	22.4 ± 1.4	15.8 ± 0.4
*E. tenuriamis*	Euc_tenu	22.4 ± 1.5	7.5 ± 0.3	27.6 ± 2.2	16.1 ± 0.6
*E. vernicosa*	Euc_vern	15.4 ± 1.1	7.5 ± 0.2	10.4 ± 1.0	15.5 ± 0.3
*E. viminalis*	Euc_vimi	27.6 ± 1.1	8.5 ± 0.2	28.0 ± 1.9	16.9 ± 0.4

In each treatment, seedling height was recorded at the start of the dehydration phase.

**Figure 1.**
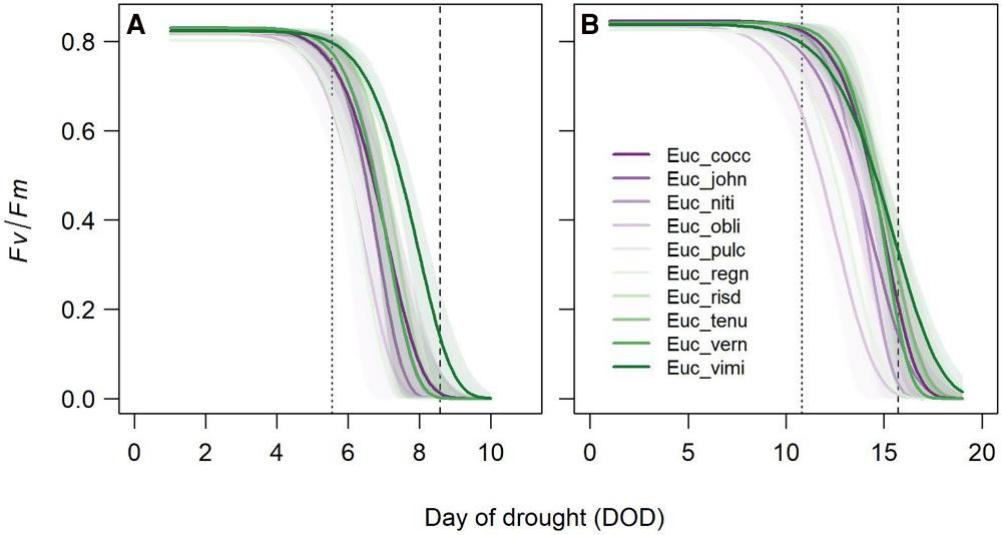
The response of *Fv/Fm* in seedlings of each species during the dry-down to death treatments. **A)** Shows the *Fv/Fm* response under warm treatment conditions. **B)** Shows the *Fv/Fm* response under cool treatment conditions. Each curve (and associated 95% CI) represents a Weibull function fitted to the pooled data for each species across trays within each treatment (see Supplementary Figs. S1 to S3 for curves fitted to individual seedlings). Dotted and dashed vertical lines indicate the day of drought associated with the visual signs of leaf wilt and shoot meristem death, respectively, averaged across seedlings of all species within each treatment.

Over the course of the dry-down phase in both treatments, *Fv/Fm* values in seedlings of each species remained near maximum (∼0.8) until the point of leaf wilt, after which they decreased, often precipitously, to low values at or near 0 (Fig. 1; Supplementary Figs. S1 to S3). Using data from 2 trays under warm treatment conditions, the start of the decline in *Fv/Fm* coincided with water potentials at ca. −2 MPa in the most sensitive species *E. obliqua* and ca. −2.8 MPa in *E. viminalis* (Supplementary Fig. S4). Under both treatments, seedling *TF*_88_ coincided closely with the time of drought leading to shoot meristem death across species (Fig. 1; Supplementary Fig. S5).

**Figure 2.**
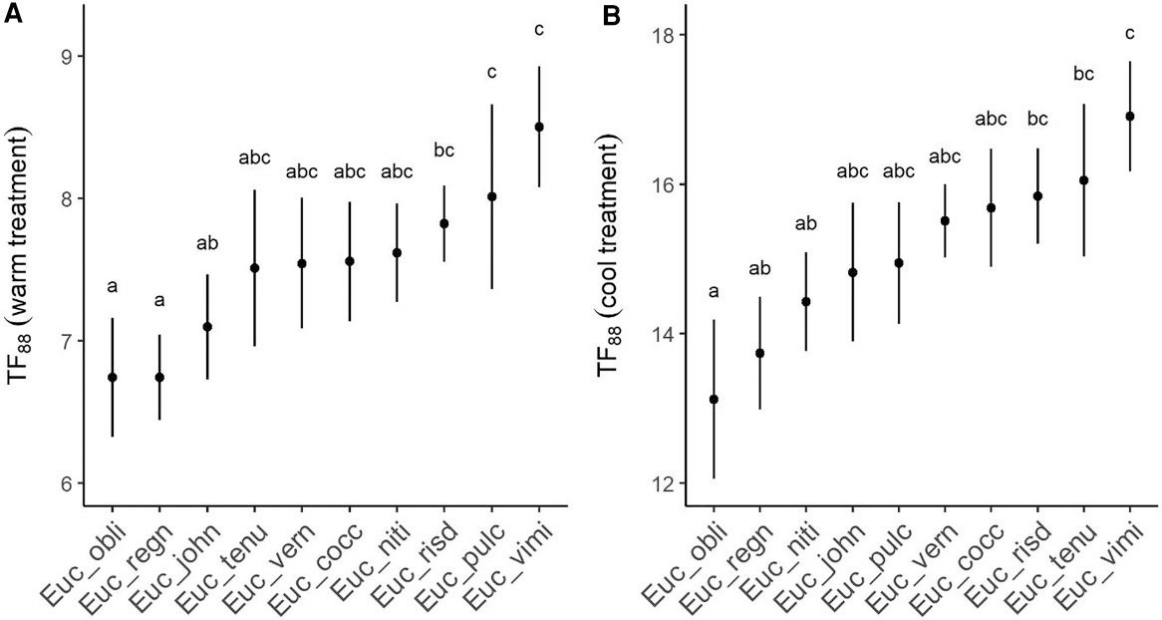
Differences among species in the time for seedling *Fv/Fm* to decline by 88% (*TF*_88_) under drought. **A)** Shows species differences under warm conditions. **B)** Shows species differences under cool conditions. Data points (solid circles) and whiskers represent species means and 95% CIs, respectively. Differences in *TF*_88_ between species in each experiment were tested using *Anova*. Significant differences (*P* ≤ 0.05) between species are denoted by different letters.

**Figure 3.**
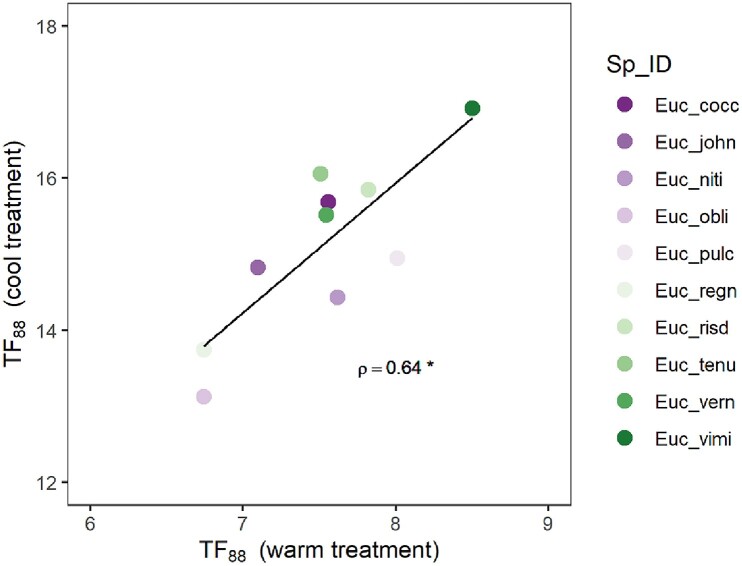
Consistency in the rank-order of species seedling drought tolerance as measured by *TF*_88_ under warm and cool temperature treatments. Spearman's rank correlation of *ρ* = 0.64. Level of significance: *P ≤ 0.05.

Linear mixed models that included a random intercept for species ID (see the “Materials and methods” section) were strongly preferred (dAIC > 40; Table 1), indicating that *TF*_88_ varied significantly among species in both the warm and cool treatments, respectively. In the warm treatment, these differences were observed despite the narrow range of mean *TF*_88_ values (6.7 to 8.5) recorded across species. Importantly, differences in *TF*_88_ were significant after accounting for differences in seedling height within and between species. Indeed, seedling height was not a significant predictor of *TF*_88_ (*Anova*: χ^2^_df__=__1_ = 0.01, *P* = 0.91; see also Supplementary Fig. S6). Under warm temperature conditions, *TF*_88_ was shortest for *E. obliqua* (6.7 d) and *E. regnans* (6.7 d) and longest for *E. pulchella* (8 d) and *E. viminalis* (8.5 d), while under cool temperature conditions, *TF*_88_ was shortest for *E. obliqua* (13.1 d) and *E. regnans* (13.7 d) and longest for *E. tenuriamis* (16.1 d) and *E. viminalis* (16.9 d; Table 2; Fig. 2). The significant positive rank-order correlation of species *TF*_88_ (*ρ* = 0.64, *P* = 0.05; Fig. 3) indicated that species rank-order of drought tolerance was consistent under warm and cool treatment conditions.

**Figure 4.**
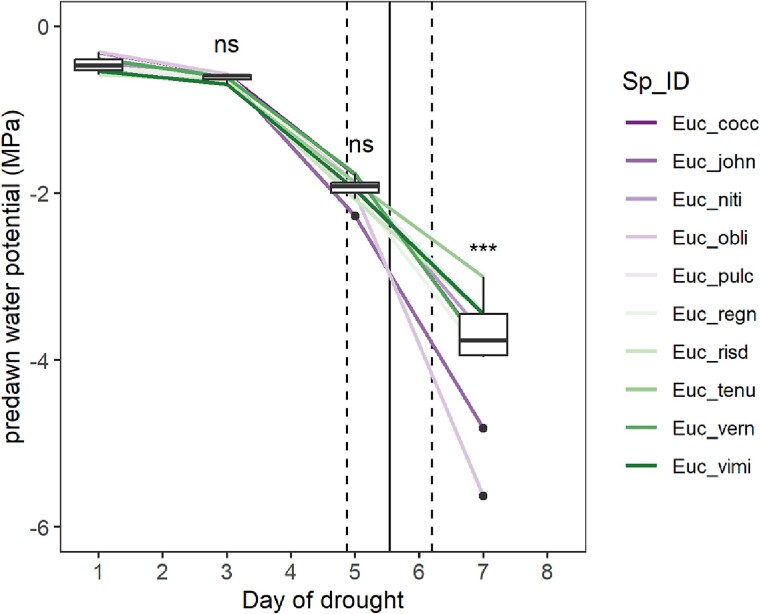
Changes in predawn plant water potential in response to soil drying measured at 4 time points in seedlings of 10 *Eucalyptus* species planted in each of 2 mixed-planting trays under warm temperature conditions. Individual colored lines represent species mean seedling responses across both trays. Box plots show the spread of species mean water potential values recorded on each measurement day, with each boxplot consisting of the center line, median; upper and lower quartiles; whiskers, 1.5 × inter-quartile range; and points, outliers. Differences in species mean predawn water potentials (*n* = 4) were tested using *Anova* at measurement dates: Day 3, 5, and 7. Levels of significance are indicated: ns = *P* > 0.05; ***P < 0.001. Vertical lines indicate the average (solid line ± Sd [dashed lines]) day of first leaf wilt recorded across individuals.

Predawn water potential decreased for all seedlings over the course of the dry-down treatment under warm temperature conditions (Fig. 4). During this time, seedling water potential did not differ significantly among species on Days 3 and 5 leading up to the point of visual signs of leaf wilt, which for most seedlings occurred between Days 5 and 6 at water potentials around −2.5 MPa. Similarity in predawn water potentials during early phase drought suggests rates of soil dehydration were relatively homogenous within trays and not impacted by differences in plant position within trays or any differences in evaporation across the soil surface. On Day 7, significant differences in plant water potential emerged between species as the seedlings of less-resistant species began show visual signs of leaf death (Fig. 4).

**Figure 5.**
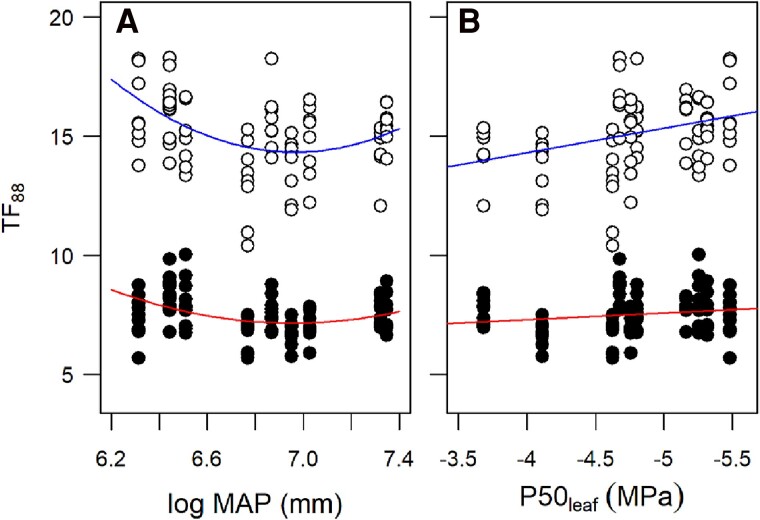
Relationships across species between seedling *TF*_88_ and climate and between seedling *TF*_88_ and adult cavitation vulnerability. **A)** Shows the relationship between *TF*_88_ and the log of MAP at each species sampling site. **B)** Shows the relationship between *TF*_88_ and species cavitation vulnerability (*P*50_leaf_) measured previously in adult foliage (Hartill et al. 2023). The best-fit quadratic functions in **A)** are shown for data from the warm (lower line; *y* = 125.7 + log[MAP] × −34.1 + log[MAP]^2^ × 2.4) and cool (upper line; *y* = 267.6 + log[MAP] × −72.7 + log(MAP)^2^ × 5.2) treatments. The best-fit linear functions in **B)** are shown for data from the warm (lower line; *y* = 6.1 + |*P*50| × 0.28) and cool (upper line; *y* = 10.3 + |*P*50| × 1.03) treatments (see *P*-values for all parameters in each model in Supplementary Table S2). In each plot, warm (filled circles) and cool (nonfilled circles) treatment conditions are indicated.

Across species, models including quadratic relationships between (log) MAP and *TF*_88_ were consistently preferred over models including only linear relationships (Supplementary Table S1 showing akaike information criterion [AIC] of all models). The best-supported model included a near significant interaction between treatment and the square of (log) MAP (χ^2^_df__=__1_ = 3.6, *P* = 0.06), suggesting distinct positive quadratic relationships between seedling *TF*_88_ and site MAP in the 2 treatments (Fig. 5A). Specifically, longer *TF*_88_ was reported in seedlings of species from either dry or wet sites, but this effect was most pronounced in the cool treatment. Importantly, these wet sites are also exposed to freezing winter temperatures. A significant interaction between treatment and adult *P*50_leaf_ (χ^2^_df__=__1_ = 7.1, *P* = 0.008) indicated that seedling *TF*_88_ was more strongly positively related to leaf cavitation vulnerability under cool treatment than warm treatment conditions (Fig. 5B).

## Discussion

Under both warm and cool treatment conditions, we detected significant differences between species in the time for seedlings to reach critical thresholds of physiological dysfunction during drought, despite their being significant differences between species in seedling size (height). Seedling size did not influence our measure of seedling drought tolerance, either across or within species, meaning that larger individuals did not use up available soil water and dehydrate more rapidly than smaller individuals. This was confirmed by our measurements of predawn water potential in seedlings under warm treatment conditions, which did not vary significantly among individuals within trays during early phase drought leading up to the expected point of incipient cavitation and associated physiological dysfunction in the most vulnerable species. Thus, the mixed-planting approach appears to overcome the size-bias present in many drought experiments where plants are grown in individual pots (Juenger and Verslues 2023). Given that rates of dehydration were similar across different sized individuals and that species rank-order of seedling drought tolerance was consistent under contrasting temperature conditions, we argue that the mixed-planting approach provides a strong basis for comparison of seedling drought tolerance across species.

### Variation in seedling drought tolerance

Our measurements of predawn water potential suggest that rates of plant dehydration were homogenous within trays while plants remained unstressed during early phase drought. Water potentials only began to diverge after visual signs of leaf wilt were recoded, and they became strongly divergent once more vulnerable species began to show strong declines in *Fv/Fm* and visual signs of death in older leaves closer to the base of the stem. We believe this divergence in water potentials and associated declines in *Fv/Fm* occurred as more vulnerable individuals reached critical levels of drought stress leading to runaway embolism and rapid plant dehydration (Tonet et al. 2023). Similar declines in *Fv/Fm* have previously been linked to thresholds of damage to water transport pathways due to embolism, as well as to mechanisms linked to photochemical damage (Brodribb et al. 2021; Fortunel et al. 2023; Tonet et al. 2023). Additionally, we observed a near 1:1 relationship across species in both treatments between *TF*_88_ and the day of drought associated with shoot meristem death. This suggests our measure of an 88% decline in *Fv/Fm* was associated with lethal drought stress, although we note that seedling recoverability following rewatering was not assessed. Taken together, these findings suggest that species differences in the time for seedlings to dehydrate to critical levels of physiological dysfunction (*TF*_88_) represent fundamental differences in species xylem cavitation resistance.

We hypothesized that variation in seedling drought tolerance across species would be correlated with variation in leaf xylem cavitation vulnerability previously measured in adult foliage (Hartill et al. 2023), assuming that local environmental filters were related to drought effect seedlings and adults similarly. This expectation was strengthened given that seedlings in the current study were grown from seed sourced from the same trees used to measure leaf cavitation vulnerability, which minimized potential errors due to within-species variability in hydraulic traits. We observed a positive relationship across species between seedling *TF*_88_ measured under cool treatment conditions and adult *P*50_leaf_ measured in field grown trees. This result points to the heritability of xylem cavitation vulnerability in eucalypts consistent with evidence of strong adaptive selection of drought tolerance traits across *Eucalyptus* species with widely contrasting rainfall distributions (Pfautsch et al. 2016; Bourne et al. 2017; Li et al. 2018; Peters et al. 2021). Furthermore, it suggests that meaningful differences in species drought tolerance in adult trees can be assessed by comparing differences in drought tolerance in seedlings, allowing for scaled-up approaches to phenotyping drought tolerant ideotypes across large numbers of individuals (Pita-Barbosa et al. 2023).

We also observed a significant quadratic relationship between *TF*_88_ and (log) MAP at each species sampling site, suggesting strong natural selection for seedling drought tolerance across the climatic range of these species, including high rainfall environments that experience winter freezing (Hartill et al. 2023). Importantly, these associations between *TF*_88_ and both adult *P*50_leaf_ and site MAP were weaker when plants were dried-down more quickly at higher temperature, suggesting that slower dehydration under cooler conditions is better for stratifying drought tolerance differences between species. Nevertheless, with an increased risk of flash drought occurring in a warming climate (Christian et al. 2021, 2023), there is still a need to examine plant physiological responses and tolerance thresholds during hot-drought events.

While we observed associations between seedling drought tolerance and both adult hydraulic vulnerability and site climate, we might expect to see even stronger relationships if seedlings of each species were allowed to adjust via phenotypic plasticity to their local climatic conditions. Plasticity in hydraulic and allocation traits is commonly observed in eucalypts in response to water deficit (Pritzkow et al. 2020; Challis et al. 2022) or growth along gradients in water availability (Zolfaghar et al. 2015a, 2015b; Smith et al. 2023), although evidence from the few studies on plasticity in xylem cavitation vulnerability is mixed. Increased xylem cavitation resistance in *Eucalyptus* has been reported in saplings of *Corymbia* (a closely related genus of eucalypt) grown under water deficit (Challis et al. 2022) and elevated temperature (Blackman et al. 2017), as well as in wild populations of *E. globulus* compared with trees of the same species from a common garden field trial (Lucani et al. 2019). However, another study observed no change in cavitation resistance in saplings of *E. obliqua* exposed to repeated drought-recovery cycles but did observe changes in plant allometry (Pritzkow et al. 2021).

### Benefits and challenges of mixed-planting drought experiments

Somewhat surprisingly, very few studies have used mixed-planting approaches to examine differences in seedling physiology and/or survivorship during drought (but see Ranney et al. 1991; Izanloo et al. 2008; Candido-Ribeiro and Aitken 2024; Moreno et al. 2024). One recent study by Candido-Ribeiro and Aitken (2024) used the approach to examine local adaptation to drought in 2 widely distributed varieties of Douglas fir (*Pseudotsuga menziesii*). In their study, the authors grew 2,176 seedlings, representing 76 populations, across 16 mixed-planting boxes, highlighting the use of mixed-planting experiments in genomics studies aimed at identifying adaptive capacity to drought in natural populations or genetic markers for improved drought tolerance in crops.

Importantly, our results suggest that a mixed-planting approach for assessing seedling drought tolerance overcomes many of the challenges and pitfalls inherent in drought experiments using plants grown in individual pots. Prominent among these challenges is the difficulty associated with controlling rates of dehydration due to size-related differences in plant water use (Juenger and Verslues 2023). When plants of the same species are grown in separate pots containing the same volume of soil under common conditions, larger individuals with greater leaf area will always dehydrate more quickly than smaller individuals during drought. To control for this, researchers typically monitor and adjust soil water content by pot weighing. Alternatively, individual plant responses to drought can be quantified and compared by measuring changes in plant water potential (MPa). However, both these methods are time consuming and physically demanding, which limits experimental scale. Instead, our findings suggest that for experiments aimed at comparing or ranking drought tolerance among seedlings, there is no need to monitor pot weight or indeed measure plant water potential when seedlings are grown together in mixed-planting trays (or pots).

To control for differences in water use among individuals in mixed-planting drought experiments, we emphasize the need to grow seedlings together in trays or pots for sufficiently long periods of time to ensure substantial root overlap between neighbors. Six weeks was sufficient to observe strongly overlapping roots in our relatively fast-growing eucalypt seedlings, although we note that the time required will vary depending on species growth rate. Importantly, we argue that levels of root entanglement achieved in our mixed-planting experiments helped homogenize plant water status (predawn water potential) among seedlings during early phase drought, despite their being significant differences in seedling size between species. In effect, the level of root entanglement in our trays suggests there was no capacity for individuals to conserve soil water locally. Indeed, rates of soil dehydration across each tray were most likely driven by the largest and/or most profligate seedlings. While we might expect to see similar rates of dehydration in these closely related species of eucalypt all with relatively similar water use strategies, the homogenizing of plant water status during early phase drought has also been demonstrated in mixed-planting experiments using divergent species (conifer vs angiosperm) strongly contrasting in water use strategy (Moreno et al. 2024).

Growing mixed-planting seedlings in a fine-textured loam with small pore size is also important for achieving homogenous rates of plant dehydration given its low vulnerability to hydraulic separation or isolation from the roots (Sperry et al. 1998; Hultine et al. 2006). Root hydraulic isolation during drought typically occurs as a function of root shrinkage, especially fine root shrinkage, and the formation of soil-air gaps that disrupts hydraulic conductivity at the root–soil interface (North and Nobel 1997; Lo Gullo et al. 1998; Rodriguez-Dominguez and Brodribb 2020; Duddek et al. 2022). Plants growing in sandy soils may be especially prone to hydraulic isolation during periods of high transpiration and drought (Koebernick et al. 2018). However, the extent to which hydraulic isolation occurs tends to vary between species and depends on whether plants are grown in fine vs coarse textured soils (Cai et al. 2021). Recent imaging and technical innovations are improving our ability to probe root hydraulic function in intact plants (Bourbia et al. 2021; Harrison Day et al. 2023). Nevertheless, there are still substantial gaps in our knowledge of how roots of different species interact with different soil types prior to and during drought. Until these gaps are filled, we assume that fine-textured loamy soils are inherently best for maintaining root–soil contact across individuals in mixed-planting drought experiments.

Taken together, the findings from this study point to mixed-planting drought experiments as a highly useful approach for screening differences in drought tolerance among seedlings. We focused our comparison of drought survivorship among seedlings during a single dry-down to death treatment. However, the approach could also be applied to a range of research questions aimed at probing genetic and phenotypic differences in plant drought tolerance over multiple drought-recovery cycles or in response to drought under different temperature conditions. Given that the approach can be scaled-up to incorporate large numbers of seedlings (see Candido-Ribeiro and Aitken 2024), mixed-planting drought experiments hold promise for increasing our capacity to both identify species risk of drought mortality and improve plant drought tolerance in both natural and agricultural systems.

## Materials and methods

### Species

For this study, we selected a group of 10 native species of the tree genus *Eucalyptus* from contrasting climates in Tasmania, Australia. This group includes species with distributions in wet-sclerophyll, dry-sclerophyll, and sub-alpine forest. Mature trees of these species vary in height and growth habit, ranging from the mallee-type *E. risdonii* and short statured species *E. vernicosa* (<2 m) through to tall emergent trees of *E. regnans* (up to 100 m). They also represent a wide range of drought tolerances, with cavitation vulnerability of adult leaves (*P*50_leaf_; MPa) ranging from −3.68 MPa in the most vulnerable species *E. nitida* to −5.48 MPa in the least vulnerable *E. tenuriamis* (Table 3; Hartill et al. 2023). In response to water deficit, eucalypts have been shown to vary across a spectrum of iso-anisohydric stomatal regulation (Li et al. 2019; Salvi et al. 2022). However, glasshouse and field studies suggest all species close stomata prior to the onset of xylem cavitation during drought (Li et al. 2019; Peters et al. 2021). Like many eucalypts and other serotinous species, seedling recruitment typically occurs after disturbance such as fire, with successful recruitment after disturbance being more likely in high rainfall years (Lamont et al. 1991).

**Table 3.** Climate variables (MAT and MAP) at each species sampling site and species mean cavitation vulnerability (*P50*_leaf_) measured on adult foliage

Species	MAT (°C)	MAP (mm)	*P50*_leaf_ (MPa)
*E. coccifera*	5.3	960	−4.80
*E. johnstonii*	9	1,127	−4.76
*E. nitida*	7.8	1,512	−3.68
*E. obliqua*	11	869	−4.62
*E. pulchella*	12	671	−5.25
*E. regnans*	9.9	1,044	−4.11
*E. risdonii*	12	627	−5.16
*E. tenuriamis*	9.6	552	−5.48
*E. vernicosa*	7.5	1,552	−5.31
*E. viminalis*	12	627	−4.67

*P50*_leaf_ measurements were sourced from a previous study (see Hartill et al. 2023).

### Experimental design

Seed of each species was collected in mid-2022 from a single population of mature trees in the field, respectively. These populations occupy sites that vary in climate, with mean annual temperature (MAT) varying between 9 and 12 °C and mean annual rainfall (MAP) varying between 552 and 1,552 mm (Table 3). Importantly, the same populations were sampled for measurements of cavitation vulnerability in adult leaves reported in a previous study (Hartill et al. 2023). Seeds were released from their capsules and placed inside paper envelopes and stored inside a cool room at 3 °C.

We ran 2 mixed-planting experiments at different times of the year under warm and cool temperatures, respectively. For the warm temperature treatment, seedlings were germinated, grown, and dehydrated during the relatively warm austral spring/summer from September to early January 2022/2023, while for the cool temperature treatment, seedlings were germinated, grown, and dehydrated during the cool austral autumn/winter from March to mid-July 2023 (see timeline schematic in Supplementary Fig. S7). For each treatment, seeds of each species were planted in individual germination tray cells (4 cm wide × 6 cm deep) containing sterilized nursery potting mix and placed inside a nonclimate-controlled glasshouse with daily temperatures ranging between 15 and 25 °C and watered daily. Seedlings germinated roughly 2 to 3 wk later in both treatments. Emergent seedlings were grown in the glasshouse for ∼5 wk before being moved outside to an open-topped enclosure and grown under ambient conditions (outside temperatures during this time are shown for both treatments in Supplementary Fig. S8). An automatic irrigation system ensured seedlings remained well-watered.

After ∼5 wk of growth under ambient conditions, representative seedlings of each species were selected for transplantation into mixed-planting trays. A total of 7 trays (Tote boxes) were used in the warm temperature treatment and a total of 4 trays were used in the cool temperature treatment, respectively (lower numbers of trays were used in the cool treatment due to lower germination success for some species). Each tray measured 40 cm (L) × 30 cm (W) × 12 cm (H) with 12 evenly spaced drainage holes. A total of 20 seedlings representing 2 individuals per species were transplanted into each tray, with seedlings spaced evenly in a 5 × 4 configuration. Each tray was conceptually divided into 2 and a single individual per species randomly assigned to a position in each half. This process was repeated for each tray so that seedling position of each species varied across trays. A total of 140 and 80 seedlings were transplanted across the 7 and 4 trays under warm and cool temperature conditions, respectively (see Supplementary Fig. S7).

A fine-textured loam soil with added slow-release fertilizer was carefully infilled between seedlings to a depth of 8 cm. We used this type of soil to maximize contact between the roots and soil during drought, as opposed to using nursey grade potting mix which typically contains coarse material and airspaces that would cause roots to become separated from the soil matrix, potentially leading to differences in dehydration rates between seedlings. Each tray was placed on a sturdy wire-mesh base and moved to a bench in the seedling enclosure and regularly watered. A spirit level was used to ensure each tray was level to prevent pooling of soil water. Seedlings were allowed to grow for an additional 5 to 6 wk to allow for the roots of each seedling to overlap with those of its neighbors (inspection of the soil at the end of the experiment indicated that this was in fact the case; see Supplementary Fig. S9). At the end of this growth phase, the height of each individual was measured.

Trays were moved back into either the nonclimate-controlled glasshouse for the warm treatment or a climate-controlled glasshouse cell for the cool treatment and leveled in position. Seedlings were watered daily and allowed to acclimate to the glasshouse conditions for 1 wk prior the start of the dry-down to death phase. The mean minimum and maximum (min/max) temperatures inside the warm treatment glasshouse during the dry-down to death phase was 16.0/26.4 °C, while the cool treatment glasshouse was set to night/day temperatures of 10/18 °C (see Supplementary Fig. S8). These temperatures resulted in contrasting dehydration times across individual seedlings, ranging from 7 to 10 d in the warm treatment and 15 to 20 d in the cool treatment.

### Dry-down to death

**Figure 6.**
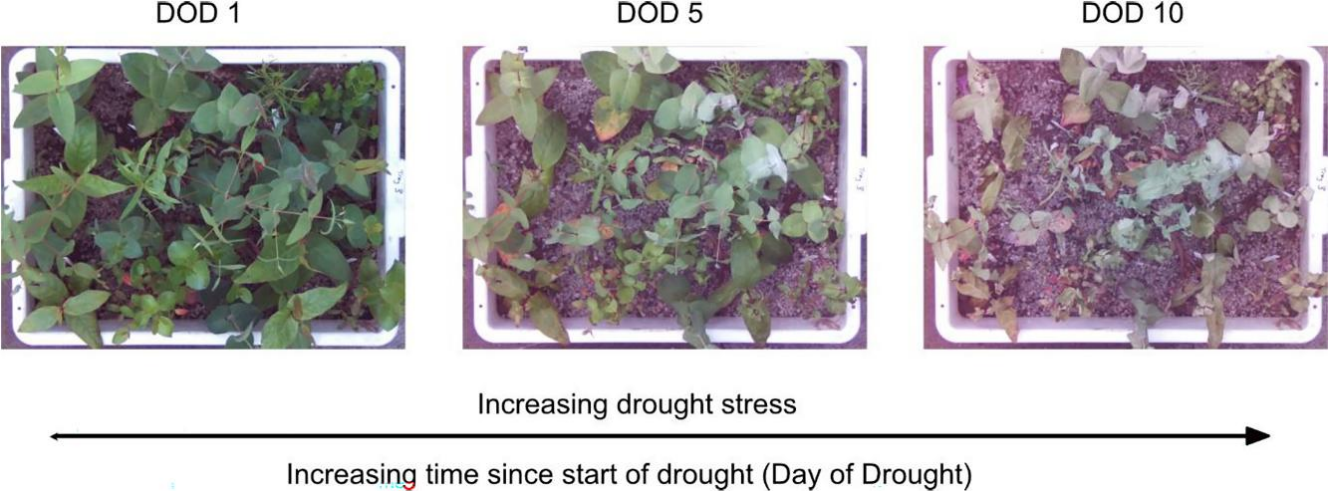
The progression of drought stress in seedlings growing together in a single tray dried-down to death over 10 d under warm treatment conditions. At day of drought (DOD) 1, all seedlings are hydrated; at DOD 5, most seedlings show signs of leaf wilt; and at DOD 10, all seedlings have reached *Fv/Fm* values <0.05 (representing a >88% decline from maximum) and have succumbed to water stress.

The dry-down to death phase started with a short drought-recovery cycle whereby seedlings in the warm and cool temperature treatments were dehydrated over the course of 4 and 8 d, respectively, to the point of leaf wilt observed in most seedlings across trays and then rewatered. This ensured individuals were drought hardened (Thomas 2009). Dry-down to death was then commenced by withholding water and allowing seedlings to use up available soil moisture and dehydrate to critical levels of drought stress associated with plant death as judged when shoot meristems became dry and brittle to touch (see Fig. 6). In both treatments, a daily score was given to seedlings in all trays based on visual and tactile signs of plant health during drought: 1 = leaves fully turgid; 2 = leaves wilted; 3 = first leaf death (typically recorded in older leaves at the base of the stem); and 4 = shoot meristem death (recorded when the meristem was dry and brittle to touch).

Dark adapted *Fv/Fm* was measured on a single upper canopy, fully expanded leaf of each seedling in each of 7 trays in the warm treatment and in each of 4 trays in the cool treatment using a Mini-Pam-II Photosynthesis Yield Analyser (Walz, Effeltrich, Germany). Importantly, these upper canopy leaves were the last to succumb to catastrophic dehydration during drought. Over the course of the dry-down phase, measurements were taken in the morning every 2nd day while plants were well hydrated and then daily following the first signs of leaf wilt. On each measurement day, seedlings were moved temporarily to a dark room located next to the glasshouse and dark adapted for 1 h. Measurements continued for all individuals in each tray until *Fv/Fm* values recorded in the most drought tolerant individual were at or near zero for 2 consecutive days.

To test the efficiency of soil water and plant water status homogenization during drought, predawn plant water potential was measured in all individuals of 2 trays under warm treatment conditions at 4 different time points (days) during early phase drought leading up to the onset of seedling leaf death in more vulnerable seedlings. At between 5 and 6 PM on the day before each measurement date, the 2 trays were moved to the dark room next to the glasshouse and leveled in position. The following morning at between 7 and 8 AM, a single leaf of each seedling was excised with a razor at the base of the petiole and immediately placed inside a humidified plastic bag and stored in a humidified esky. A small side-shoot was sampled for *E. viminalis* with small sessile leaves. The 2 trays of seedlings were then returned to the glasshouse. The water potential of each sample was measured using a Scholander-type pressure chamber.

### Statistical analysis

We characterized species seedling drought tolerance in terms of their ability to maintain *Fv/Fm*, expressed as a percentage of maximum values, over the course of the dry-down to death phase under warm and cool treatment conditions, respectively. To do this, we fitted a Weibull model function using the *fitplc* package in R to the *Fv/Fm* vs time (days) since last watering (T) data recorded for each individual seedling, with the final watering day defined as *T* = 0 (see Supplementary Fig. S1 for an example curve fitted to an individual seedling and Supplementary Figs. S2 and S3 for curves fitted to all seedlings of each species within each treatment). From these functions, we calculated the *T* associated with an 88% decline in *Fv/Fm* (*TF*_88_). While lower levels of percentage *Fv/Fm* decline have been reported to coincide with critical levels of stress and recoverability in response to and following drought (e.g. Brodribb et al. 2021; Fortunel et al. 2023), we used an 88% percent decline in *Fv/Fm* on the basis that it would more likely coincide with catastrophic hydraulic failure and plant death (Tonet et al. 2023). Indeed, following the dry-down to death phase, we observed close correspondence, especially under cool treatment conditions, between *TF*_88_ and the day of drought associated with shoot meristem death (Supplementary Fig. S5). Furthermore, the rank-order of species *TF*_88_ within each treatment was strongly conserved when species drought tolerance was characterized using lower thresholds of *Fv/Fm* decline set at 12% (Spearman’s rank correlation [rho, *ρ*]; *ρ* > 0.69, *P* < 0.05) and 50% (*ρ* > 0.97, *P* < 0.001), respectively (Supplementary Fig. S10). Differences in seedling height among species, trays, and treatment conditions at the start of the drought phase were tested using a linear mixed model, fitting seedling height as the response variable, treatment as a fixed effect, and both tray ID and species ID as random intercepts. To test for effects of species identity, treatment, and seedling height on seedling drought tolerance, we ran a linear mixed model, fitting *TF*_88_ as the response variable, treatment, and seedling height as fixed effects, and both species ID and tray ID as random intercepts. We compared AIC between models with or without random intercepts. To make multiple comparisons of seedling T_88_ between species under warm and cool treatments, respectively, we also ran a linear mixed model using species ID and seedling height as fixed effects and tray as a random variable. Post hoc pairwise comparisons of *TF*_88_ between species were assessed using the *emeans* function with a “Tukey” adjustment.

To test for cross-species relationships between seedling *TF*_88_ in each treatment condition and each of (log) site MAP and species mean adult leaf cavitation vulnerability, we refit the linear mixed model substituting fixed effects of either (log) MAP or adult *P*50_leaf_ for seedling height. To evaluate evidence for nonlinear relationships between these variables and seedling *TF*_88_, we compared AIC between models containing linear and quadratic terms (Supplementary Table S1) and present only fitted lines from preferred models.

For all linear mixed models, the significance of fixed effects was tested using Type II Wald χ^2^ tests with the *Anova* function from the *car* R package. The significance of random effects (tray and species ID) was tested via model comparison by sequentially dropping random effects and comparing the AIC of candidate models. In 2 individual trays under warm treatment conditions, differences in species mean leaf water potentials were tested on Days 3, 5, and 7 since last watering, respectively, using *Anova* with tray included as a random variable. A Spearman’s rank correlation was used to test for consistency in the rank-order of species mean height and *TF*_88_ between treatments, respectively. All statistics were performed in R version 4.3.0 (see Supplementary Text S1).

## Supplementary Material

kiae632_Supplementary_Data

## Data Availability

The data underlying this article will be shared on reasonable request to the corresponding author.

## Supplementary data

The following materials are available in the online version of this article.

**Supplementary Figure S1.** An example of the response of *Fv/Fm* measured in a single seedling of *E. coccifera* during drought.

**Supplementary Figure S2.** Plots showing the response of *Fv/Fm* measured in seedlings of each species across trays under warm temperature conditions.

**Supplementary Figure S3.** Plots showing the response of *Fv/Fm* measured in seedlings of each species across trays under cool temperature conditions.

**Supplementary Figure S4.** The response in *Fv/Fm* relative to the response in leaf water potential recorded for 2 species under warm treatment conditions.

**Supplementary Figure S5.** Correspondence across species between *TF*_88_ and the day of drought associated with shoot meristem death under cool and warm treatments.

**Supplementary Figure S6.** The relationship between *TF*_88_ and height recorded across seedlings of each species under cool and warm treatments.

**Supplementary Figure S7.** Diagrams of the experimental design, including planting position within a single tray, tray number within each treatment, and a timeline of key phases during the experiment.

**Supplementary Figure S8.** Differences in temperature conditions between the cool and warm treatments during seedling growth and final dry-down phases.

**Supplementary Figure S9.** Evidence of strong root entanglement within a single tray.

**Supplementary Figure S10.** Correspondence across species between *TF*_88_ and *TF*_12_ and between *TF*_88_ and *TF*_50_ measured under cool warm treatments.

**Supplementary Table S1.** Comparison of AIC for linear mixed models containing either linear or quadratic terms of the relationship between seedling *TF*_88_ and site MAP and adult *P*50_leaf_, including an interaction with treatment (_int).

**Supplementary Table S2.** Analysis of deviance table showing χ^2^ and significance values for each of the parameters in the preferred models of the relationship between *TF*_88_ and both MAP and adult *P*50_leaf_, respectively.

**Supplementary Text S1.** *R_code*: Code used in the analysis described in the study.

## Dive Curated Terms

The following phenotypic, genotypic, and functional terms are of significance to the work described in this paper:

root AmiGo: PO:0009005

seed AmiGo: PO:0009010

water CHEBI: CHEBI:15377
