# Supplementary material for: All together now: A mixed-planting experiment reveals adaptive drought tolerance in seedlings of 10 Eucalyptus species
Source: Plant Physiol. 2024 Nov 29;197(1):kiae632. doi: 10.1093/plphys/kiae632 (PMC11663711; doi:10.1093/plphys/kiae632)
Supplement: kiae632_Supplementary_Data [file kiae632_supplementary_data.zip › supp code.docx]

Supplementary material (R-code) for the manuscript:

**All together now: A mixed-planting experiment reveals adaptive drought tolerance in seedlings of 10 *Eucalyptus* species**

Chris J Blackman, Ben Halliwell and Tim J Brodribb

Supplementary Text S1. *R_code*: Code used in the analysis described in the study.

library(emmeans);library(dplyr);library(doBy);library(multcomp);library(fitplc);library(lme4)

library(ggplot2);library(corrplot);library(Hmisc); library(cowplot); library(RColorBrewer); library(gridExtra)

### Files

Drydown_RAW <- read.csv("Euc_seedling_drydown_RAW.csv")

Euc_means <- read.csv("Euc_seedling_means.csv")

# Individual traits

Indiv_drought_resp <- read.csv("Indiv_drought_resp.csv")

Indiv_drought_resp$Sp_ID <- as.factor(Indiv_drought_resp$Sp_ID)

summary(Indiv_drought_resp)

### example seedling with a Weibull function fitted to Fv/Fm response to drought

Drydown_RAW_FvFm <- subset(Drydown_RAW, !is.na(Y..II.))

FvFm_Euc_cocc_1 <- subset(Drydown_RAW_FvFm, Sp_ID=='Euc_cocc' & rep_ID=='1' & Treat=='warm' & allo=='main')

FvFm_Euc_cocc_fit <- fitcond(FvFm_Euc_cocc_1, varnames=c(K="Y..II.", WP="DOD"), WP_Kmax = 2, model="Weibull", x=88, nboot=1000)

getPx(FvFm_Euc_cocc_fit, x=12)

getPx(FvFm_Euc_cocc_fit, x=50)

getPx(FvFm_Euc_cocc_fit, x=88)

plot(FvFm_Euc_cocc_fit, plotPx=T, plotci=F, px_ci="none", main=expression(italic("Eucalyptus coccifera rep1")),

xlab="Day of Drought", ylab="Fv/Fm")

### Comparison of height between treatments

h_fit <- lme4::lmer(Hght_cm ~ Treat + (1|tray) + (1|Sp_ID), Indiv_drought_resp, REML = F) ### final model !!!

h_fit %>% summary

# testing significance of fixed effects

car::Anova(h_fit)

# testing significance of random effects

h_fit_tray <- lme4::lmer(Hght_cm ~ Treat + (1|tray), Indiv_drought_resp, REML = F)

h_fit_Sp <- lme4::lmer(Hght_cm ~ Treat + (1|Sp_ID), Indiv_drought_resp, REML = F)

h_fit_no_rand <- lm(Hght_cm ~ Treat, Indiv_drought_resp)

anova(h_fit, h_fit_Sp, h_fit_tray, h_fit_no_rand)

### Comparison of TF88 among species

# both treatments together

fit <- lme4::lmer(TF88 ~ Treat + Hght_cm + (1|tray) + (1|Sp_ID), Indiv_drought_resp, REML = F) ### final model !!!

fit %>% summary

car::Anova(fit)

fit_tray <- lme4::lmer(TF88 ~ Treat + Hght_cm + (1|tray), Indiv_drought_resp, REML = F) ### final model !!!

fit_Sp <- lme4::lmer(TF88 ~ Treat + Hght_cm + (1|Sp_ID), Indiv_drought_resp, REML = F) ### final model !!!

fit_no_rand <- lm(TF88 ~ Treat + Hght_cm, Indiv_drought_resp) ### final model !!!

anova(fit, fit_Sp, fit_tray, fit_no_rand)

### Comparison of LWP among species at each measurement date (DOD) in two trays

LWP <- subset(Drydown_RAW, Treat=='warm' & allo=='LWP')

LWP_1 <- subset(Drydown_RAW, !is.na(MPa))

tray2_7 <- subset(LWP_1, subset = tray %in% c(2,7))

tray2_7 <- filter(tray2_7, !DOD %in% c(4,6,8))

LWP_DOD3 <- subset(tray2_7, DOD=="3")

LWP_DOD5 <- subset(tray2_7, DOD=="5")

LWP_DOD7 <- subset(tray2_7, DOD=="7")

fitDOD3 <- lme4::lmer(MPa ~ Sp_ID + (1|tray), LWP_DOD3)

fitDOD3 %>% summary

car::Anova(fitDOD3)

### Comparison of species rank order for DOD88 and height, respectively.

# DOD88_warm vs DOD88_cool

cor.test(x=Euc_means$DOD88_warm, y=Euc_means$DOD88_cool, method = 'spearman', exact=T)

# height

cor.test(x=Euc_means$Hght_warm, y=Euc_means$Hght_cool, method = 'spearman')

### Analysis of relationship between seedlings DOD@PLCF88 and MAP and adult P50

# MAP (individual observations)

# match MAP values from Euc_means data

Indiv_drought_resp$MAP <- log(Euc_means[match(Indiv_drought_resp$Sp_ID, Euc_means$Sp_ID),]$MAP)

# fit linear and quadratic models

fit_map_lin <- lmer(TF88 ~ Treat + MAP + (1|tray) + (1|Sp_ID), Indiv_drought_resp, REML = F)

fit_map_lin_int <- lmer(TF88 ~ Treat * MAP + (1|tray) + (1|Sp_ID), Indiv_drought_resp, REML = F)

fit_map_quad <- lmer(TF88 ~ Treat + MAP + I(MAP^2) + (1|tray) + (1|Sp_ID), Indiv_drought_resp, REML = F)

fit_map_quad_int <- lmer(TF88 ~ Treat * MAP + Treat * I(MAP^2) + (1|tray) + (1|Sp_ID), Indiv_drought_resp, REML = F)

# summaries

fit_map_lin %>% summary()

fit_map_lin_int %>% summary()

fit_map_quad %>% summary()

fit_map_quad_int %>% summary() # final (preferred) model

# significance tests

Anova(fit_map_lin)

Anova(fit_map_lin_int)

Anova(fit_map_quad)

Anova(fit_map_quad_int) # final (preferred) model

# compare models

anova(fit_map_lin, fit_map_lin_int, fit_map_quad, fit_map_quad_int)

# P50 (individual observations)

# match P50 values from Euc_means data

Indiv_drought_resp$P50_adult <- abs(Euc_means[match(Indiv_drought_resp$Sp_ID, Euc_means$Sp_ID),]$P50_adult)

# subset treatment

warm <- subset(Indiv_drought_resp, Treat=='warm')

cool <- subset(Indiv_drought_resp, Treat=='cool')

# fit linear and quadratic models

fit_P50_lin <- lmer(TF88 ~ Treat + P50_adult + (1|tray)+ (1|Sp_ID), Indiv_drought_resp, REML = F)

fit_P50_lin_int <- lmer(TF88 ~ Treat * P50_adult + (1|tray)+ (1|Sp_ID), Indiv_drought_resp, REML = F)

fit_P50_quad <- lmer(TF88 ~ Treat + P50_adult + I(P50_adult^2) + (1|tray)+ (1|Sp_ID), Indiv_drought_resp, REML = F)

fit_P50_quad_int <- lmer(TF88 ~ Treat * P50_adult + Treat * I(P50_adult^2) + (1|tray) + (1|Sp_ID), Indiv_drought_resp, REML = F)

# summaries

fit_P50_lin %>% summary()

fit_P50_lin_int %>% summary() # final (preferred) model

fit_P50_quad %>% summary()

fit_P50_quad_int %>% summary()

# significance tests

Anova(fit_P50_lin)

Anova(fit_P50_lin_int) # final (preferred) model

Anova(fit_P50_quad)

Anova(fit_P50_quad_int)

# compare models

AIC(fit_P50_lin, fit_P50_lin_int, fit_P50_quad, fit_P50_quad_int)
