# Supplementary material for: All together now: A mixed-planting experiment reveals adaptive drought tolerance in seedlings of 10 Eucalyptus species
Source: Plant Physiol. 2024 Nov 29;197(1):kiae632. doi: 10.1093/plphys/kiae632 (PMC11663711; doi:10.1093/plphys/kiae632)
Supplement: kiae632_Supplementary_Data [file kiae632_supplementary_data.zip › suppdata.docx]

**All together now: A mixed-planting experiment reveals adaptive drought tolerance in seedlings of 10 *Eucalyptus* species**

Chris J Blackman, Ben Halliwell and Tim J Brodribb


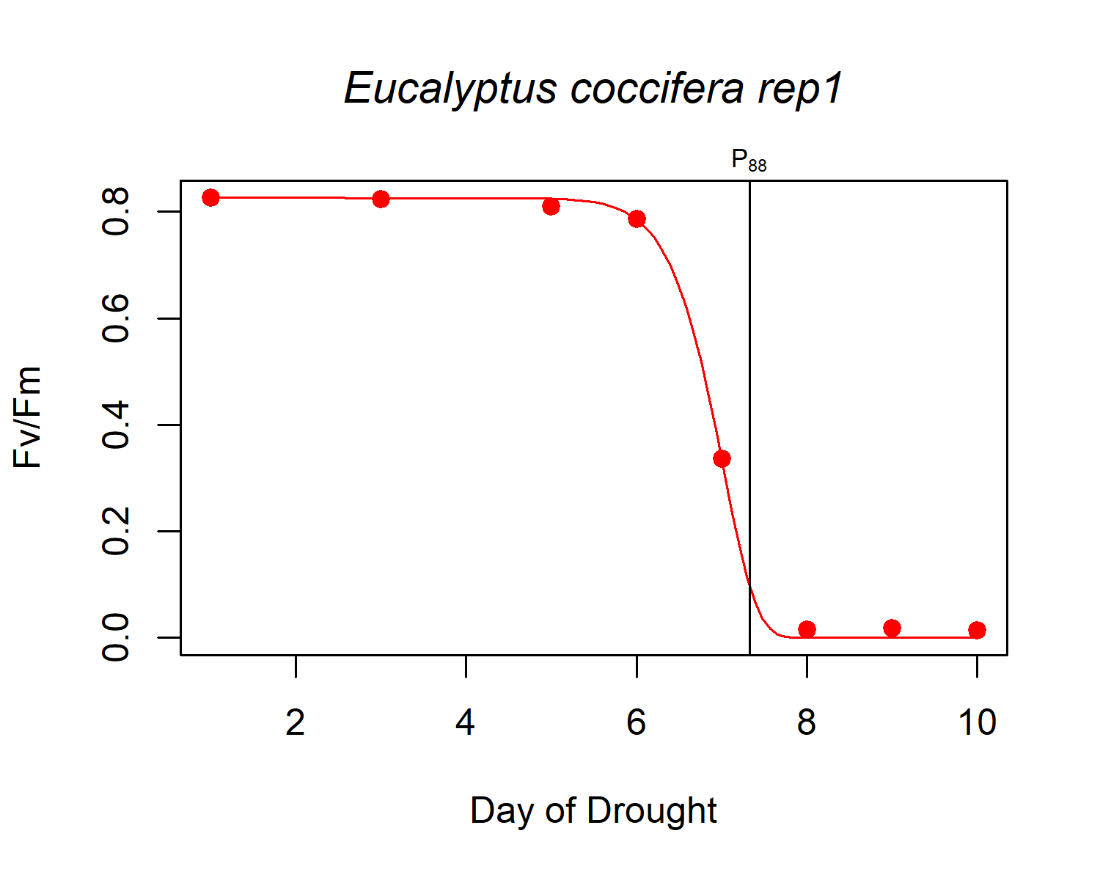


Supplementary Figure S1. An example of the response of *Fv/Fm* measured in a single seedling of *Eucalyptus coccifera* during drought. The curve is a Weibull function fitted using the *fitplc* package in R. The vertical black line indicates the day of drought associated with an 88% reduction in *Fv/Fm* (TF_88_).


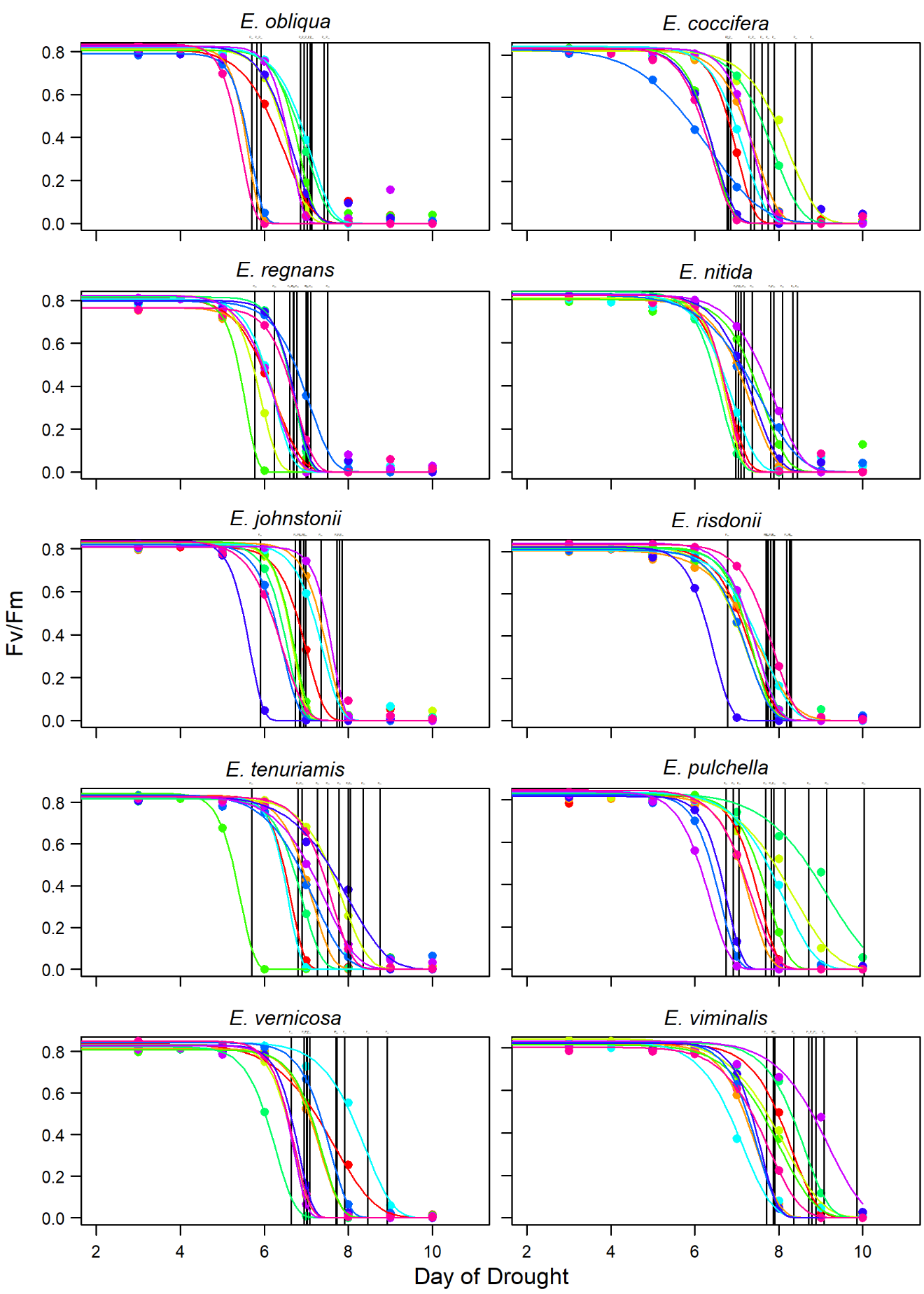


Supplementary Figure S2. Plots showing the response of *Fv/Fm* measured in seedlings of each species across trays under warm temperature conditions. Within each plot, each curve is a Weibull function fitted to data measured in a single individual using the *fitplc* package in R, while different coloured data points and lines indicate different individuals. Solid vertical lines indicate the day of drought associated with an 88% reduction in *Fv/Fm* (TF_88_) recorded for each individual, respectively.


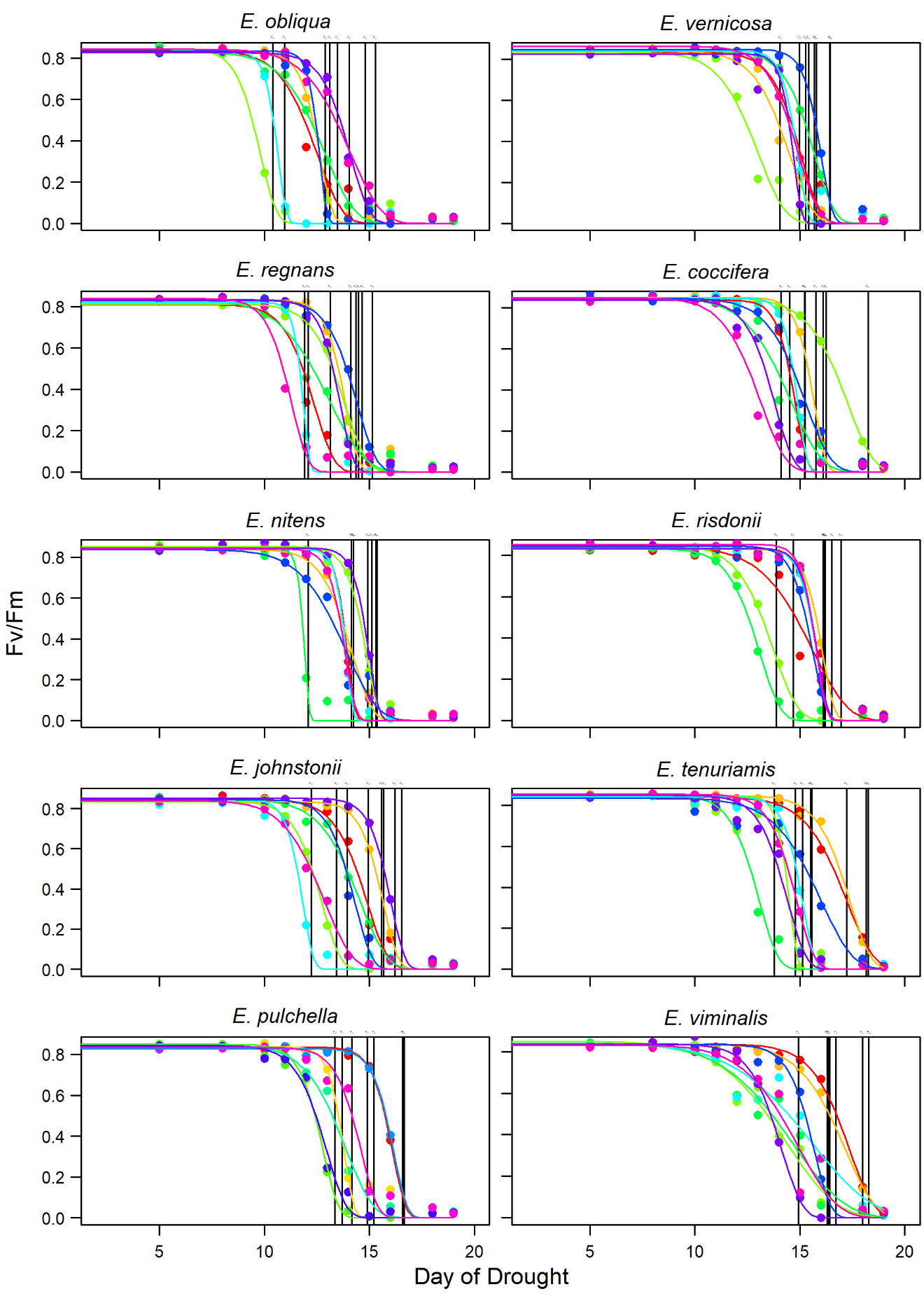


Supplementary Figure S3. Plots showing the response of *Fv/Fm* measured in seedlings of each species across trays under cool temperature conditions. Within each plot, each curve is a Weibull function fitted to data measured in a single individual using the *fitplc* package in R, while different coloured data points and lines indicate different individuals. Solid vertical lines indicate the day of drought associated with an 88% reduction in *Fv/Fm* (TF_88_) recorded for each individual, respectively.


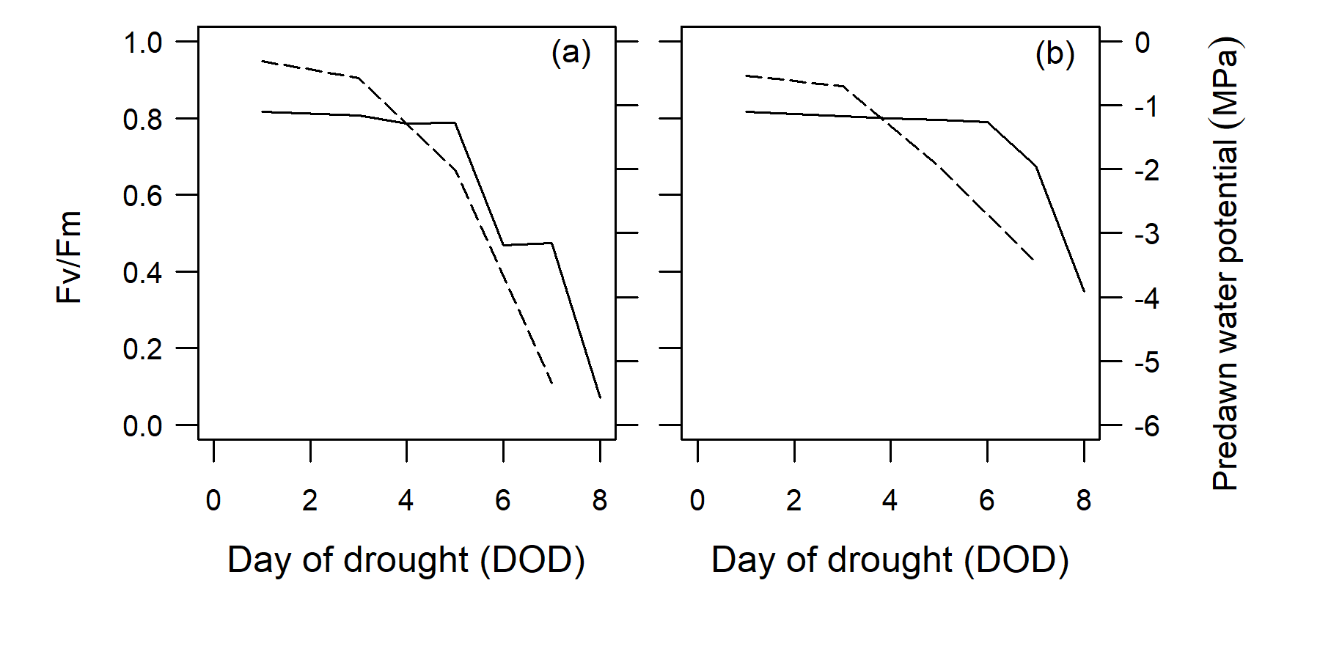


Supplementary Figure S4. The response in *Fv/Fm* relative to the response in leaf water potential recorded for two species under warm treatment conditions. Plots of the response of *Fv/Fm* (solid lines) and predawn plant water potential (dashed lines) during drought in the most vulnerable species *E. obliqua* (a) and least vulnerable species *E. viminalis* (b) to drought, based on differences in TF_88_. These data were drawn from seedlings from two trays under warm treatment conditions.


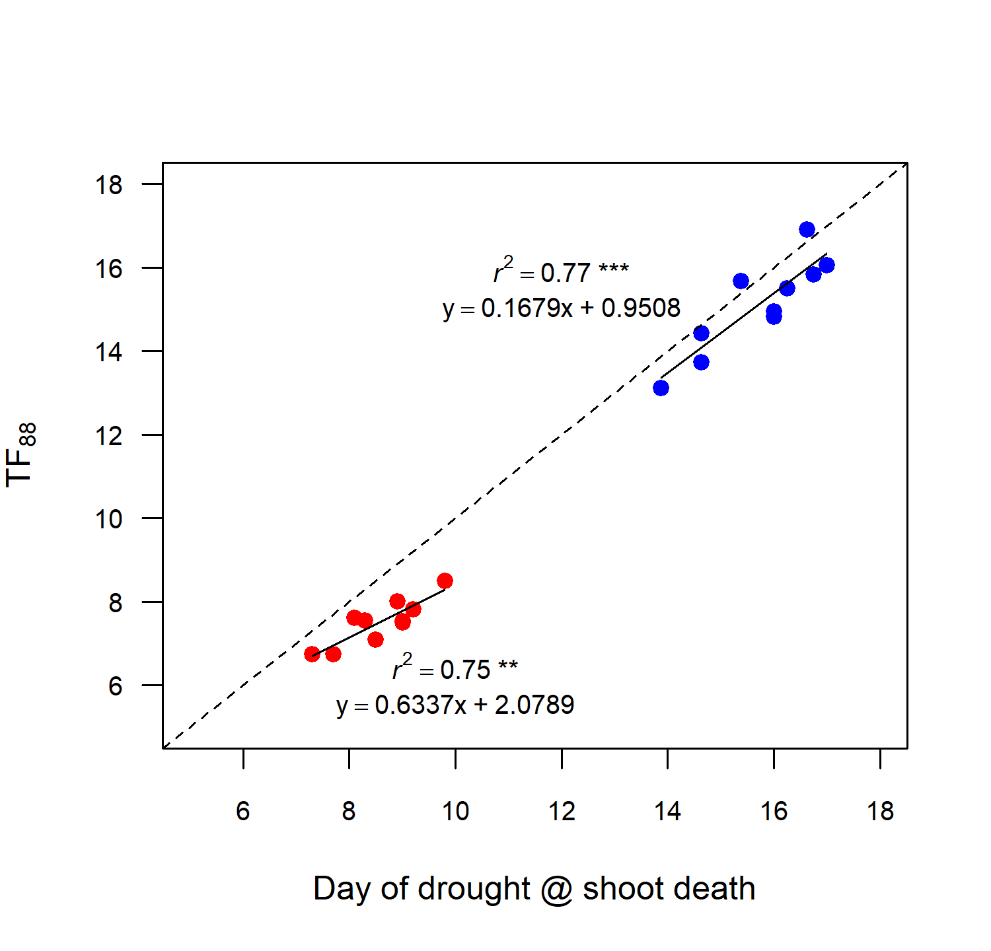


Supplementary Figure S5. Correspondence across species between *TF*_88_ and the day of drought associated with shoot meristem death under cool and warm treatments. Plot showing near 1:1 correspondence between the day of drought associated with 88% loss in *Fv/Fm* (TF_88_) and the day of drought associated with the first signs of shoot meristem death, calculated as the mean for each species. Red and blue data points represent warm and cool treatment conditions.


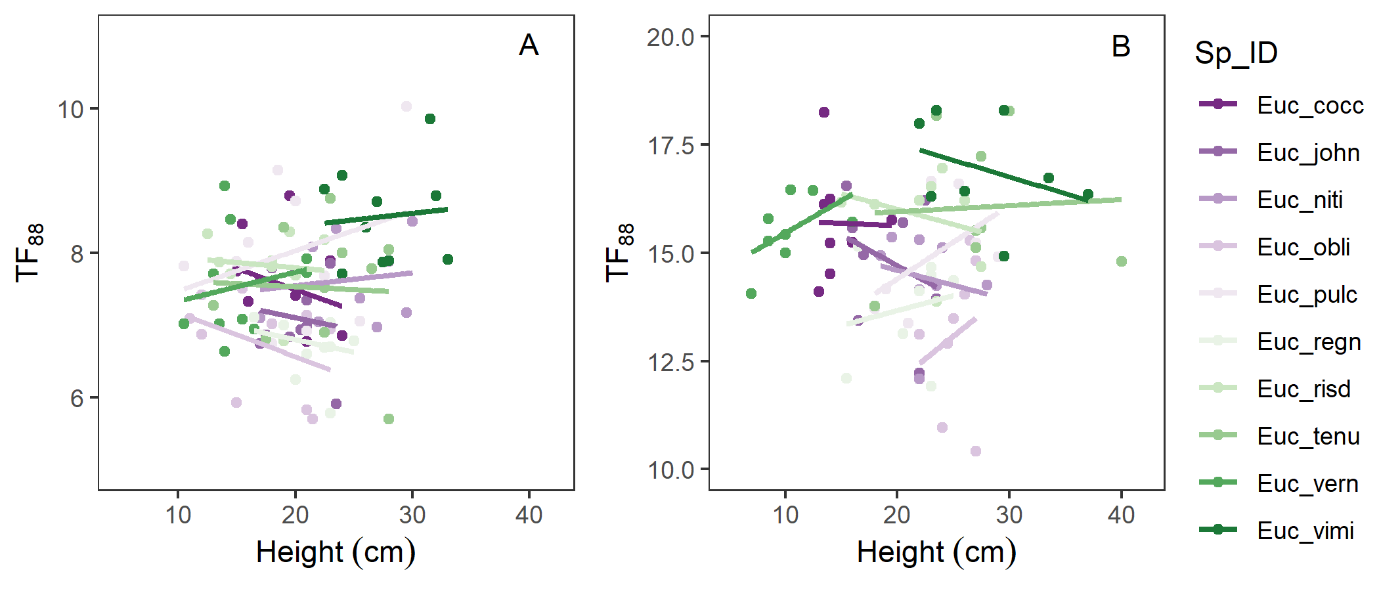


Supplementary Figure S6. The relationship between *TF*_88_ and height recorded across seedlings of each species under cool and warm treatments. Plots showing relationships between TF_88_ and height across individuals of each species under warm (plot A) and cool (plot B) treatment conditions, respectively. In both treatments, the effect of height on TF_88_ was not significant across species.


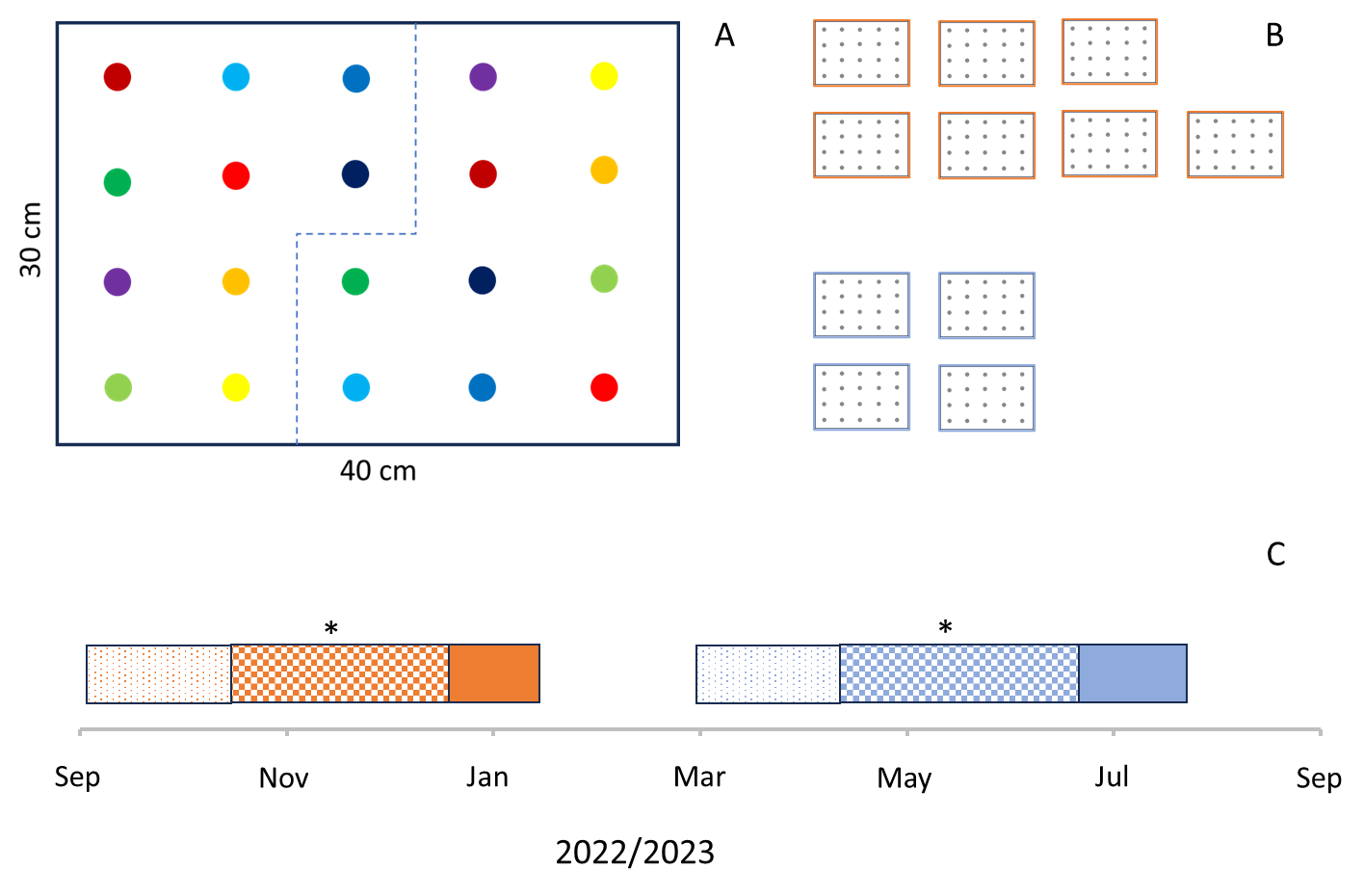


Supplementary Figure S7. Diagrams of the experimental design, including planting position within a single tray, tray number within each treatment, and a timeline of key phases during the experiment. Plot A, a schematic showing the placement of seedlings within a single tray. Each tray contained a total of 20 seedlings representing 2 seedlings per 10 species, spaced evenly in a 5 x 4 configuration. The planting area of each tray was divided in two (dashed line) and a single individual of each species (different coloured symbols) randomly assigned to a planting position within each half. This process was repeated for each tray, respectively, so that seedlings of each species were likely to be in different positions across trays. Plot B, a total of seven trays (in orange) were used under warm temperature conditions and a total of four trays (in blue) were used under cool treatment conditions. Plot C, a timeline chart showing the timing and duration of key stages of each experiment under warm (orange) and cool (blue) treatment conditions. For both treatments, the stippled area represents germination and early seedling growth within the glasshouse; the checked area represents seedling growth outside under ambient conditions (also see Fig S2); the solid-fill area represents the final dry-down to death. Asterisks represent the time when seedlings were transplanted from individual germination cells into the mixed-planting trays.


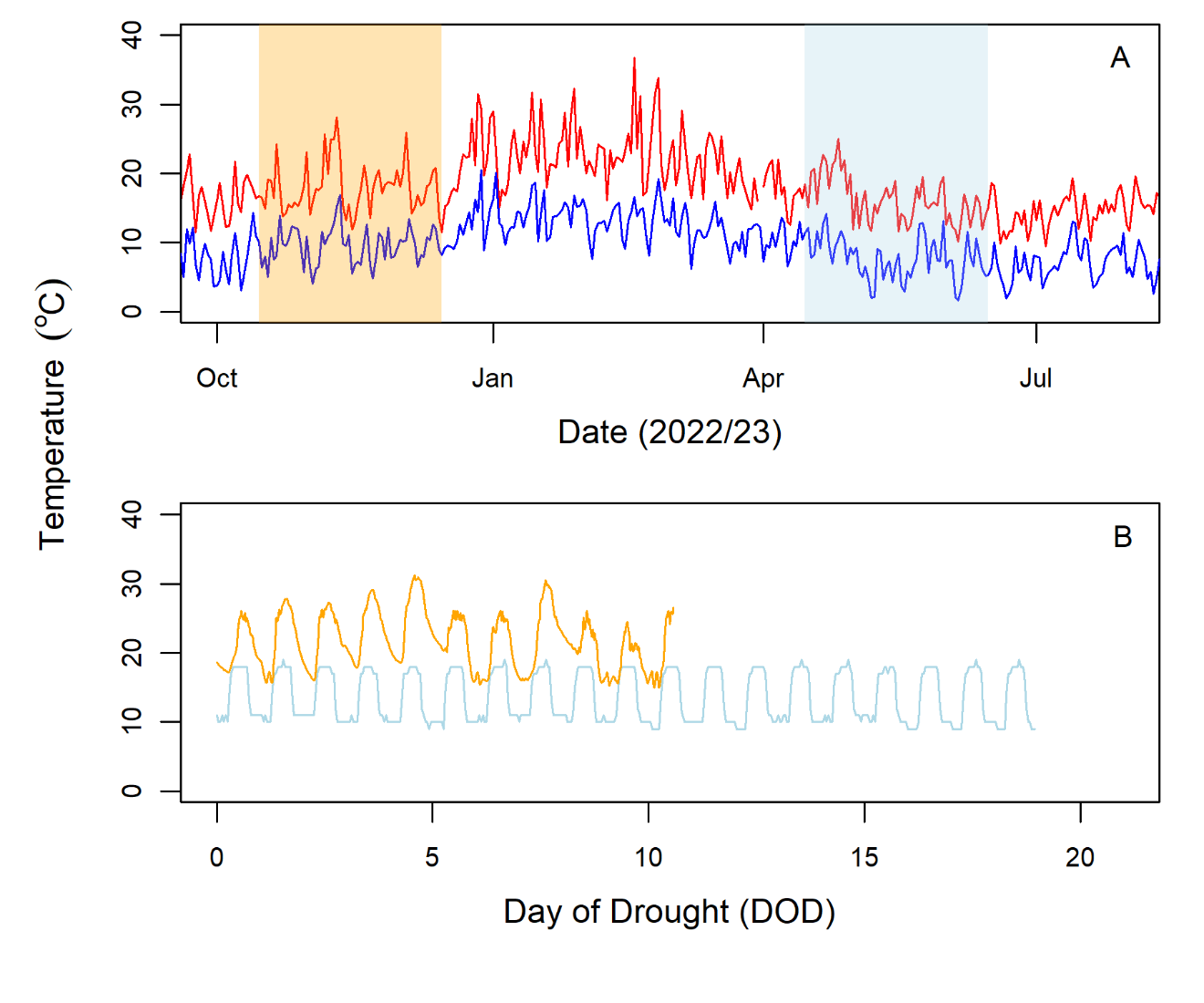


Supplementary Figure S8. Differences in temperature conditions between the cool and warm treatments during seedling growth and final dry-down phases. Plot A, Maximum (red line) and minimum (blue line) outside temperatures recorded from a weather station (Hobart, Ellerslie Rd; http://www.bom.gov.au/) nearby to where seedlings were grown in the current study. The orange and light blue shaded areas represent the time seedlings were grown outside prior to being moved back into the glasshouse for each dry-down to death phase under warm and cool treatments, respectively. Plot B, Diurnal glasshouse temperatures recorded during the dry-down to death phase under the warm (orange line) and cool (light blue line) treatments conditions, respectively.


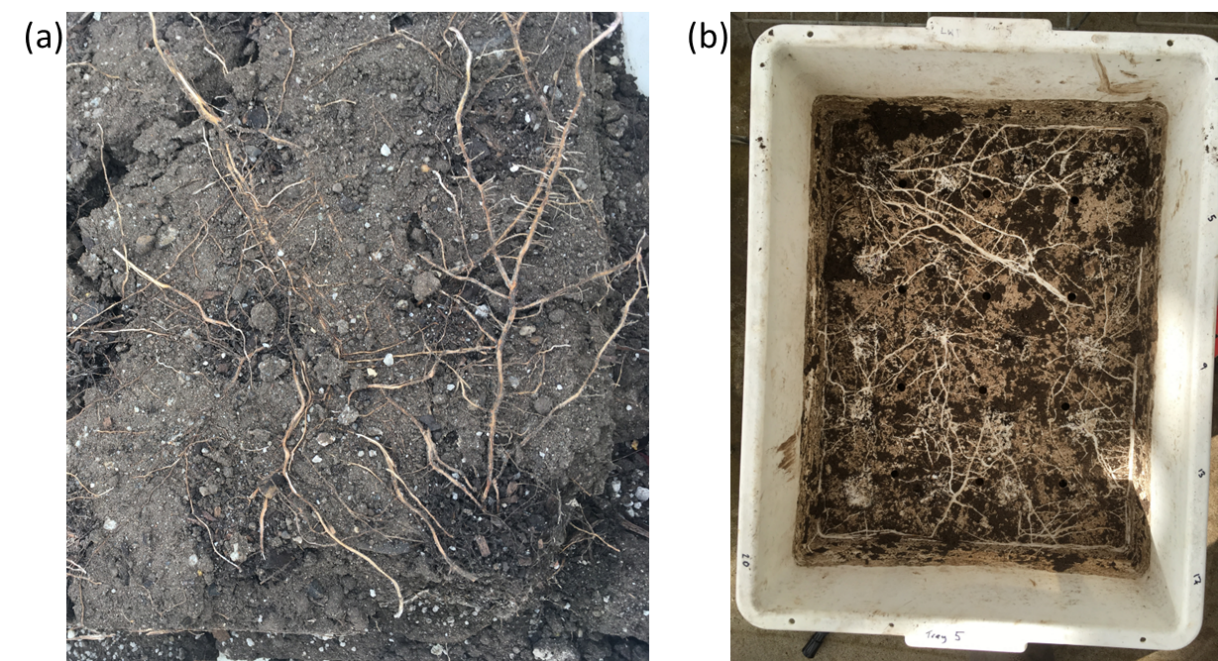


Supplementary Figure S9. Evidence of strong root entanglement within a single tray. (a) A representative image of the underside of the soil from a tray of mixed-planting seedlings following drought. The image shows substantial root overlap between neighbours. (b) An imprint from the bottom of the same tray after the soil was removed showing a network of overlapping roots.


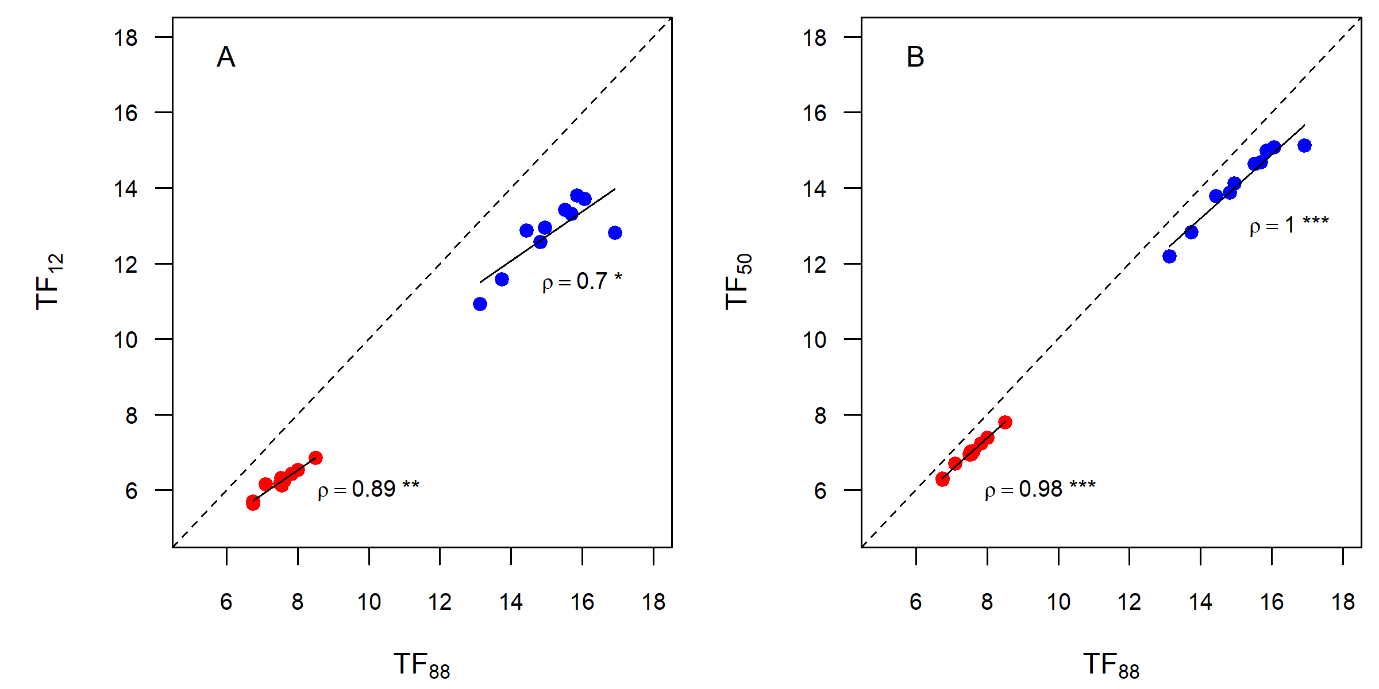


Supplementary Figure S10. Correspondence across species between *TF*_88_ and *TF*_12_ and between *TF*_88_ and *TF*_50_ measured under cool warm treatments. Plots showing strong consistency in species rank-order between the time (T) for seedlings to reach thresholds of Fv/Fm (F) decline at 88% (*TF*_88_) and both 12% (*TF*_12_; plot A) and 50% (*TF*_50_; plot B), under warm (red symbols) and cool (blue symbols) treatments, respectively. Spearman’s rank correlation (rho; *ρ*) are shown. Level of significance: * = *P* ≤ 0.05; ** = *P* ≤ 0.01; *** = *P* ≤ 0.0001.

Supplementary Table S1. Comparison of AIC for linear mixed models containing either linear or quadratic terms of the relationship between seedling TF_88_ and site MAP and adult *P50*_leaf_ including an interaction with treatment (_int). Best-fit models (bolded) were identified with the lowest AIC score.

| Model | AIC |  | Model | AIC | |
| --- | --- | --- | --- | --- | --- |
| *TF_88_ ~ MAP* |  |  | *TF_88_ ~ P50_leaf_* (P50) | |  |
| MAP_fit_lin | 552.4 |  | P50_fit_lin | 552.4 | |
| MAP_fit_lin_int | 551.5 |  | P50_fit_lin_int | **547.4** | |
| MAP_fit_quad | 550.6 |  | P50_fit_quad | 554.3 | |
| MAP_fit_quad_int | **548.0** |  | P50_fit_quad_int | 551.3 | |

Supplementary Table S2. Analysis of deviance table showing Chisq and significance values for each of the parameters in the preferred models of the relationship between TF_88_ and both MAP and adult *P50*_leaf_, respectively.

| Model | Chisq | Df | P-value |  |
| --- | --- | --- | --- | --- |
| *TF_88_ ~ MAP* |  |  |  |  |
| Treatment | 1839.9 | 1 | < 2e-16 |  |
| MAP | 4.91 | 1 | 0.027 |  |
| I(MAP^2^) | 4.74 | 1 | 0.029 |  |
| Treatment:MAP | 3.74 | 1 | 0.053 |  |
| Treatment:IMAP^2^ | 3.60 | 1 | 0.058 |  |
|  |  |  |  |  |
| *TF_88_ ~ P50_leaf_* (P50) |  |  |  |  |
| Treatment | 1831.6 | 1 | <2.2e-16 |  |
| P50 | 2.40 | 1 | 0.121 |  |
| Treatment:P50 | 7.10 | 1 | 0.008 |  |
