# Supplementary material for: All together now: A mixed-planting experiment reveals adaptive drought tolerance in seedlings of 10 Eucalyptus species
Source: Plant Physiol. 2024 Nov 29;197(1):kiae632. doi: 10.1093/plphys/kiae632 (PMC11663711; doi:10.1093/plphys/kiae632)
Supplement: kiae632_Supplementary_Data [file pp.00822.2024-s06.pdf › pp.00822.2024-s06.pdf]

Plant Physiology®

All together now: mixed-planting experiment reveals adaptive drought tolerance in seedlings of ten Eucalyptus species

Christopher Blackman, Ben Halliwell, and Timothy Brodribb

Review Timeline:

Submission Date:	31-May-2024
Editorial Decision:	01-Sep-2024
Revision Received:	04-Nov-2024
Accepted:	10-Nov-2024

01-Sep-2024

Dr. Christopher J Blackman
University of Tasmania
Hobart, TAS
Australia

RE: All together now: mixed-planting experiment reveals adaptive drought tolerance in seedlings of ten Eucalyptus species

Dear Dr. Blackman:

We have received reviews of your manuscript, which you may view at the end of this letter. On the basis of the advice received and my own evaluation, I believe that, pending revision, this article may ultimately be acceptable for publication in Plant Physiology.

Please address the concerns raised by the two reviewers. In addition, my principle concern is that I feel the paper missed the opportunity to contrast the current data with some single pot measurements - therein to demonstrate the advantage of the multi-pot approach. I realize that it is not possible to add this now, without repeating everything - but I encourage the authors to discuss further the difficulties in interpreting single-pot dry down experiments and how they can be problematic/misleading.

When you are ready to submit your revision, click the below link and find the red arrow under your Author Tasks heading for this revision submission.

If you want to visit this version of the manuscript, you may click the link below and then click on the folder that reads "Post Decision Manuscripts".

Link Not Available

When uploading the revised version of this article, please be sure to include as complete an answer as possible to the referees' criticisms during the first round of reviews in the "response to reviewers" section.

Plant Physiology considers a revision for up to 60 days after a manuscript has been provisionally accepted; limited extension of this timeframe is at the discretion of the Monitoring Editor. A reminder notice will be sent to you 2 weeks before the deadline, at which time you may choose to contact the editorial office to request an extension; if so, please provide a justification for your request. After the expiration of this deadline, the article would need to be submitted as a resubmission and would be handled as such.

Thank you very much for giving us an opportunity to review this work. I look forward to receiving the next version.

Sincerely,

Editor
Plant Physiology

Want to add this revision deadline to your calendar? Click below!

-- Reviewer comments:
Reviewer #1 (Comments for the Author):

This study examines the value of using a mixed planting experiment to classify the drought tolerance of 10 Eucalyptus species seedlings. Drought tolerance of species mixed all together were characterized by the day when Fv/Fm decline by 88% (DOD@PLCF88) under warm and cool conditions.

The mains results of the study are that :
1) mixing species in tray allows to homogenize their dehydration, and hence allows to compare temporally their drought tolerance.
2) DOD@PLCF88 gives same ranking of species drought tolerance in both warm and cool conditions.
3) DOD@PLCF88 correlates well with P50 values obtained on adult leaf of same species only under the cool temperature experiment.
With the intensity and frequency of droughts increasing worldwide, numerous forest diebacks have been observed. It is hence crucial to characterize the drought tolerance of tree species to predict their mortality risk and select species that can withstand drier conditions. The methodology used to rank the drought tolerance of species in this study is simple to implement and may be of great interest to help select species (all plant categories) that would best tolerate aggravated water stress.

Globally, I found the paper clear and well written. The major comments I have to raise on this study concerns the added value of using a mixed planting experiment to homogenize plant dessication over time. As the soil surface of the trays were not covered, how can we be sure that plant dehydration is not mainly caused by soil evaporation ? Hence, I wonder if assembling the same number of plants of the same species (with tangled roots) wouldn't have produced the same results ?
Is it not possible to characterize drought tolerance of species based on the relationship between DOD@PLCF88 and predawn water potential ? If yes, it is also a way to avoid the problem of dehydration time according to species/height...
Finally, even if there is no differences between species predawn water potential until the DOD5, how can we ensure that the soil is uniformly dry on DOD7, when differences in predawn water potentials between species are significant. By the way, what causes variations of species water potentials on DOD7 ? Discussion on this point is lacking.

###Specific comments
L.26 : What happens in the warm temperature conditions ? Please specify it.
L.83-99 : A scheme would help to better understand the experiment (the same applies to the experimental design section of the Mat & Meth).
L.97 : The first hypothesis seems strange: is it a question of distinguishing the warm-temperature experiment from the cool-temperature one ? If yes, it would be more understandable to say so.
L114 : Table 1. It would be interesting to add information about stomatal regulation of species.
L1140 : Why use 7 trays for the experiment in warm conditions versus only 4 for the experiment in cool conditions ?
L.145 : Is the position of each plant the same in each tray, or does it differ from one tray to the next ? To specify.
L.166-168 : How long to reach first wilt ? Please, indicate it.
L.170 : « Visible signs of shoot meristem death », what are these signs ?
L.171 : The two trays used to measured predawn water potential are always the same or differed according to the date of measurement ? To be indicated.
L.193-195 : In the Figure 5, it would be interesting to distinguish the grey lines between species using the same color code as in figure 4. Why didn't you make the same graph for the other cool treatment ? Please, add it.
L.197-209 : Please, specify the hours exactly.
L216 : This is not shown in fig. S6, but rather in fig. S7. Are there any significant differences between the DOD@PLCF88 ~ DOD@shoot death of both temperature conditions ? Indeed, under warm conditions, the relationship doesn't really fit the 1:1 line. Could you indicate the exact equation (ax+b) of DOD@PLCF88 ~ DOD@shoot in both temperature conditions and the pvalue of the fit ?
L.255-257 : I do not understand the test made. Please, could you reformulate the sentence.
L.336 : Fig 6 : Please, could you indicate the equation and pvalue of fit for both temperature treatment.
L.429-431 : Not really sure that cool temperature is more realistic method to used as warmer climate is expected worldwide. P50 represent only one trait accounting for drought tolerance of species. Stomatal closure and cuticular stomatal conductance also play crucial roles in preventing hydraulic failure. HSM and newly SMRI_P50 (https://doi.org/10.1093/jxb/erad352) should be more robust traits to used for identifying drought tolerance of species.

Reviewer #2 (Comments for the Author):

Blackman et al presents a study that used seedlings of 10 eucalyptus species to attempt to quantify and rank the species ability to survive drought using a unique type of common planting where the seedlings were grown together in a common planter and dried down. Leaf health was quantified using Fv/Fm. They found that there is a relationship between seedling survival length and adult leaf p50. I apricate the inclusion of the cool vs warm experiments, and that the order of fv/fm88 matches between experiments. The cool appears to be much more useful in stratifying the vulnerability of the plants likely due to the slower water loss rates. The use of the single pot multiple species is an interesting way to try and investigate fine differences in plant susceptibility to drought.

The term DOD@PLCF88 seems very clunky and confusing. PLC I believe refers to percent loss of conductance but that does not appear to be what is being measured here. The authors used a change in Fv/Fm.

Did the authors observe any edge effects from there experiment? I would guess that plants placed closer to the edge might have a more limited ability to root since they would immediately encounter the end of the tray. How was the effect of plant placement controlled for?

Figure 6: I am a little confused by panel A in figure 6 it indicates that the x-axis is MAP but the scale is 6.2-7.4. I am assuming this is a mix up with the MAT. This should be fixed in this figure and throughout the paper.

I am concerned with the use of the range of 0.8-0 for Fv/Fm I was under the impression that Fv/Fm numbers lower than 0.6 indicate the plant is under significant stress have the number all the way down to 0 been shown to have a meaningful relationship with plant stress and recovery? Did the authors find the point at which Fv/Fm fell to bellow 0.6 differed at all between species? This is especially true with the Fig S3 where the representative Fv/Fm curve only has a single point between 0.8 and 0.

Did the authors consider rewatering the trays to see if any species recovered? Not perhaps the present leaves but reshoots from lower buds.

The following is our response to both editor and reviewer comments. In each case, we describe how we have addressed the relevant concern or suggestion.

RE: All together now: mixed-planting experiment reveals adaptive drought tolerance in seedlings of ten Eucalyptus species

Dear Dr. Blackman:

We have received reviews of your manuscript, which you may view at the end of this letter. On the basis of the advice received and my own evaluation, I believe that, pending revision, this article may ultimately be acceptable for publication in Plant Physiology.

Please address the concerns raised by the two reviewers. In addition, my principle concern is that I feel the paper missed the opportunity to contrast the current data with some single pot measurements - therein to demonstrate the advantage of the multi-pot approach. I realize that it is not possible to add this now, without repeating everything - but I encourage the authors to discuss further the difficulties in interpreting single-pot dry down experiments and how they can be problematic/misleading.

Author response: We thank the editor for the opportunity to revise our manuscript for publication in Plant Physiology. In response to the editor's comment above, we have added a new paragraph in the Discussion that describes some of the pitfalls and problems of running drought experiments using individual pots (Ln364-376). Furthermore, having made these changes we restructured the Discussion and now define two sections of text: 1) an initial section on variation in drought tolerance observed in our study on eucalypts, and 2) the benefits and challenges of running mixed-planting drought experiments. We have added the relevant subheadings and moved and rearranged major parts of the text in the Discussion to logically fit within this structure. We feel, these changes have improved logic and clarity in the discussion of our findings and their implications to further research in the field.

…

Reviewer #1 (Comments for the Author):

This study examines the value of using a mixed planting experiment to classify the drought tolerance of 10 Eucalyptus species seedlings. Drought tolerance of species mixed all together were characterized by the day when Fv/Fm decline by 88% (DOD@PLCF88) under warm and cool conditions.
The mains results of the study are that :
1) mixing species in tray allows to homogenize their dehydration, and hence allows to compare temporally their drought tolerance.
2) DOD@PLCF88 gives same ranking of species drought tolerance in both warm and cool conditions.
3) DOD@PLCF88 correlates well with P50 values obtained on adult leaf of same species only under the cool temperature experiment.
With the intensity and frequency of droughts increasing worldwide, numerous forest diebacks have been observed. It is hence crucial to characterize the drought tolerance of tree species to predict their mortality risk and select species that can withstand drier conditions. The methodology used to rank the drought tolerance of species in this study is simple to implement and may be of great interest to help select species (all plant categories)

that would best tolerate aggravated water stress.

Globally, I found the paper clear and well written. The major comments I have to raise on this study concerns the added value of using a mixed planting experiment to homogenize plant dessication over time. As the soil surface of the trays were not covered, how can we be sure that plant dehydration is not mainly caused by soil evaporation ? Hence, I wonder if assembling the same number of plants of the same species (with tangled roots) wouldn't have produced the same results ?

Author response: We thank the reviewer for identifying this potential effect. In our study we did not examine whether plant dehydration was driven primarily via transpiration or evaporation – it was likely to be both. However, even if soil evaporation was the main source of water loss from trays, we believe the rank order of species drought tolerance would be the same – as long as rates of soil evaporation was relatively even across the tray. We believe this was the case given that we observed homogenous predawn water potentials among seedlings within individual trays during early phase drought. We have added text describing this in the results section (Ln271-273).

Is it not possible to characterize drought tolerance of species based on the relationship between DOD@PLCF88 and predawn water potential ? If yes, it is also a way to avoid the problem of dehydration time according to species/height...

Author response: By using seedlings with low numbers of leaves we were only able to collect a limited amount of water potential data. The data we did collect suggests that the relationship between Fv/Fm ~ LWP falls apart as water potentials decrease beyond critical thresholds and leaves begin to dehydrate catastrophically. We believe this decoupling between Fv/Fm and LWP is a sampling artefact and resulted from differences in the progression of leaf death within individual seedlings. For most individuals, leaves closer to the base of the plant, which we typically sampled for LWP, begun to catastrophically dehydrate earlier than the leaves in the upper part of the plant that we used to measure Fv/Fm. We describe this progression of leaf death starting in lower leaves (Ln173, Ln307) and how we limited measurements of Fv/Fm to the healthiest remaining fully expanded leaves closest to the shoot meristem (Ln177-178).

Finally, even if there is no differences between species predawn water potential until the DOD5, how can we ensure that the soil is uniformly dry on DOD7, when differences in predawn water potentials between species are significant. By the way, what causes variations of species water potentials on DOD7 ? Discussion on this point is lacking.

Author response: We thank the review for identifying this important discussion point. We assumed that soils were uniformly dry on day 7 and that the observed differences in predawn water potential are the result of differences in seedling drought tolerance. On day 7, the more vulnerable seedlings had already crossed critical thresholds and had begun to catastrophically dehydrate, while more tolerant seedlings remained within their tolerance levels. We expand our discussion on these points in the revised paper (Ln302-318).

###Specific comments
L.26 : What happens in the warm temperature conditions ? Please specify it.

Author response: We now state (Ln23-24) that 'No significant differences in water potentials were detected among seedlings dehydrated under warm conditions prior to leaf wilt.'

L.83-99 : A scheme would help to better understand the experiment (the same applies to the experimental design section of the Mat & Meth).

Author response: We thank the reviewer for their suggestion and agree that some aspects of the MM were unclear. We have now drawn up a schematic in Supplementary Fig S1 to help readers understand i) how we went about assigning plants to different positions within trays, ii) the number of trays used in the warm and cool temperature treatments, respectively, and iii) the timeline for when plants were germinated, grown and dehydrated under the warm and cool treatment conditions. We have also amended substantial sections of text to more clearly describe what we did (especially Ln122-134).

L.97 : The first hypothesis seems strange: is it a question of distinguishing the warm-temperature experiment from the cool-temperature one ? If yes, it would be more understandable to say so.

Author response: We agree that the first question/hypothesis does not logically fit within this sentence. We have removed it and instead describe this test of consistency in the rank-order of drought tolerance between treatments rather than proposing it as a hypothesis (Ln89-91).

L114 : Table 1. It would be interesting to add information about stomatal regulation of species.

Author response: Although we do not have species specific data to include in Table 1, we provide new text about stomatal regulation in eucalypts (Ln109-112):

"In response to water deficit, eucalypts have been shown to vary across a spectrum of iso-anisohydric stomatal regulation (Salvi et al., 2022, Li et al., 2019). However, glasshouse and field studies suggest all species close stomata prior to the onset of xylem cavitation during drought (Li et al., 2019, Peters et al., 2021)."

L1140 : Why use 7 trays for the experiment in warm conditions versus only 4 for the experiment in cool conditions ?

Author response: It was due to lower germination success. We now state that (Ln138-139) "lower numbers of seedlings were used in the cool treatment due to lower germination success for some species."

L.145 : Is the position of each plant the same in each tray, or does it differ from one tray to the next ? To specify.

Author response: No. Plant position was randomly assigned in each half of each tray, respectively. This meant that seedlings of all species were in a different position across trays. We now clarify this in the text (Ln141-145).

L.166-168 : How long to reach first wilt ? Please, indicate it.

Author response: We have added detail that it took around 4 and 8 days for plants to reach leaf wilt in the warm and cool treatments, respectively (Ln166).

L.170 : « Visible signs of shoot meristem death », what are these signs ?

Author response: We have added that shoot meristem death was recorded when the meristem was dry and brittle to touch (Ln174).

L.171 : The two trays used to measured predawn water potential are always the same or differed according to the date of measurement ? To be indicated.

Author response: We have deleted this sentence and provide more details in the section on water potential measurements (Ln184-193). Importantly, we include details that describe

how we measured water potentials in all individuals in each of the two trays on each measurement date.

L.193-195 : In the Figure 5, it would be interesting to distinguish the grey lines between species using the same color code as in figure 4. Why didn't you make the same graph for the other cool treatment ? Please, add it.

Author response: We thank the reviewer for this suggestion. have modified Figure 5 to distinguish species responses by colour. We did not measure LWP in the cool treatment due to a lower number of seedlings in play (low germination success for some species) and because we did not want to significantly reduce leaf area in the seedlings.

L.197-209 : Please, specify the hours exactly.

Author response: We have now specified the hours.

L216 : This is not shown in fig. S6, but rather in fig. S7. Are there any significant differences between the DOD@PLCF88 ~ DOD@shoot death of both temperature conditions ? Indeed, under warm conditions, the relationship doesn't really fit the 1:1 line. Could you indicate the exact equation (ax+b) of DOD@PLCF88 ~ DOD@shoot in both temperature conditions and the pvalue of the fit ?

Author response: Thanks for identifying the mistake, we have now corrected the misnamed text. We have also added the p-value and regression equation in both temperature conditions in Fig S7.

L.255-257 : I do not understand the test made. Please, could you reformulate the sentence.

Author response: We have rephrased the beginning of this sentence to make its meaning clearer (Ln243-246).

L.336 : Fig 6 : Please, could you indicate the equation and pvalue of fit for both temperature treatment.

Author response: We have now included the equation for each of the quadratic fits in Fig 6 plot A and linear fits in plot B. We also include a supplementary table S2 listing the p-values for each of the parameters in each of the preferred models, respectively.

L.429-431 : Not really sure that cool temperature is more realistic method to used as warmer climate is expected worldwide.

Author response: We have deleted the term 'realistic' and now more simply refer to the cool treatment as being more useful in stratifying species drought tolerance. However, in the Discussion we also comment on the need for future research to consider the impact of hot-drought events under a warming climate (Ln339-341).

"Nevertheless, with an increased risk of flash drought occurring in a warming climate (Christian et al., 2021, Christian et al., 2023), there is still a need to examine plant physiological responses and tolerance thresholds during hot-drought events."

P50 represent only one trait accounting for drought tolerance of species. Stomatal closure and cuticular stomatal conductance also play crucial roles in preventing hydraulic failure. HSM and newly SMRI_P50 (https://doi.org/10.1093/jxb/erad352) should be more robust traits to used for identifying drought tolerance of species.

Author response: We agree stomatal closure and gmin are important for species specific responses to drought. However, we did not measure either stomatal closure or minimum conductance because they shouldn't effect the outcome (ranking) of species drought tolerance when grown and dehydrated together in mixed-plant trays, assuming that rates of soil dehydration are homogenous within trays – which they were. We have added some text about this in the revised MS (Ln75-77, Ln271-273).

Reviewer #2 (Comments for the Author):

Blackman et al presents a study that used seedlings of 10 eucalyptus species to attempt to quantify and rank the species ability to survive drought using a unique type of common planting where the seedlings were grown together in a common planter and dried down. Leaf health was quantified using Fv/Fm. They found that there is a relationship between seedling survival length and adult leaf p50. I apricate the inclusion of the cool vs warm experiments, and that the order of fv/fm88 matches between experiments. The cool appears to be much more useful in stratifying the vulnerability of the plants likely due to the slower water loss rates. The use of the single pot multiple species is an interesting way to try and investigate fine differences in plant susceptibility to drought.

The term DOD@PLCF88 seems very clunky and confusing. PLC I believe refers to percent loss of conductance but that does not appear to be what is being measured here. The authors used a change in Fv/Fm.

Author response: We used DOD@PLCF$_{88}$ because a similar term is used in a previous study relating to the water potential at a certain % loss in Fv/Fm (e.g., Fortunel et al 2023). However, we agree that our use of DOD@PLCF$_{88}$ is clunky. We now define and use throughout the MS a much shorter and easier to read term (TF$_{88}$) for the time (T) to an 88% decline in Fv/Fm (F$_{88}$).

Did the authors observe any edge effects from there experiment? I would guess that plants placed closer to the edge might have a more limited ability to root since they would immediately encounter the end of the tray. How was the effect of plant placement controlled for?

Author response: We acknowledge the reviewer's concern that root growth in seedlings planted closer to the edge might have been impacted by the proximity of the side of the pot (tray). To account for this, we randomised seedling placement within trays (see Ln141-145), noting that most seedlings in a 5 x 4 configuration are in fact 'edge' plants. Importantly, we believe this potential edge effect did not alter species drought tolerance ranking given that water potential measurements were similar among all individuals within trays during early phase drought.

Figure 6: I am a little confused by panel A in figure 6 it indicates that the x-axis is MAP but the scale is 6.2-7.4. I am assuming this is a mix up with the MAT. This should be fixed in this figure and throughout the paper.

Author response: Thanks for pointing this out. The x-axis is the log of site MAP. We have added this detail to the Figure, caption and main text.

I am concerned with the use of the range of 0.8-0 for Fv/Fm I was under the impression that Fv/Fm numbers lower than 0.6 indicate the plant is under significant stress have the number

all the way down to 0 been shown to have a meaningful relationship with plant stress and recovery? Did the authors find the point at which Fv/Fm fell to bellow 0.6 differed at all between species? This is especially true with the Fig S3 where the representative Fv/Fm curve only has a single point between 0.8 and 0.

Author response: We acknowledge that lower thresholds of Fv/Fm decline have been reported in the literature. To address this concern we also calculated the time for Fv/Fm to decline by 12% (TF_{12}) and 50% (TF_{50}). Species rank order between TF_{88} and both *TF_{12} and TF_{50} was strongly conserved within each treatment using a spearman rank correlation (Ln222-225; Fig S8). Thus, it didn't really matter which metric was used for ranking species drought tolerance. As we have argued in the text, we used TF_{88} because it has been associated with critical levels of stem cavitation and plant death. Indeed, the day of drought associated with meristem death strongly corresponded with an 88% loss in FvFm in our study species, although we also acknowledge that recoverability following rewatering was not assessed (Ln202-208 in the Methods, Ln307-318 in the Discussion).*

Did the authors consider rewatering the trays to see if any species recovered? Not perhaps the present leaves but reshoots from lower buds.

Author response: We agree it would have been useful to know if any of the plants resprouted from the base and to know if our measure of physiological dysfunction represented lethal levels of water stress. However, this was not possible to control in a mixed-planting experiment where different species reached these thresholds at different times. We acknowledge that we did not measure recoverability in the text.

10-Nov-2024
Dr. Christopher J Blackman
University of Tasmania
Hobart, TAS
Australia

MSID: PP2024-RA-00822R1
MS TITLE: All together now: mixed-planting experiment reveals adaptive drought tolerance in seedlings of ten Eucalyptus species

Dear Dr. Christopher Blackman:

I am pleased to inform you that your revised manuscript "All together now: mixed-planting experiment reveals adaptive drought tolerance in seedlings of ten Eucalyptus species" has been accepted for publication in Plant Physiology. Your manuscript will be checked for consistency with journal formatting and image standards by one of our Science Editors, who will contact you shortly. They may have a number of queries and suggestions for you. Please keep in mind that they work to increase the online visibility of your article and its attraction for readers from the widest of backgrounds. You will have the opportunity to review any revisions, and I hope you will work constructively with them to get the most out of your research. You will also have the opportunity to review all changes when you receive the author proofs for your article.

Once validated for publication, your article will be sent to a Science Editor for a final review of your manuscript and source files. After your final files are uploaded, your article will appear in the next available issue and the online Preview version of your article will be posted after your signed Copyright and Checklist forms and final source files are received.

Please be sure to include the following in your manuscript file:
Harvard style citations
List of all author contributions and funding information at the beginning of manuscript after the titles and author list.
List all supplemental materials associated with the article, with titles, at the end of the ms, before the references and acknowledgments section.
Complete Figure Legends for all figures appearing in the article listed before the Literature Cited.

LICENSE INFORMATION Shortly after your paper is sent to production, you will receive an email from our publisher's author support team, **SciPris**, with information on article processing charges, as well as the types of Open Access license you would be able to purchase. The accepted version of your manuscript will not appear on our Advance Articles page until the license is selected and signed, so please look out for an email from SciPris. For more information see the Article Publication Fees section of our author guidelines: https://academic.oup.com/plcell/pages/General-Instructions

Finally, we encourage your submission of artwork for the journal cover. If there is an image or illustration related to your paper that you would like to have considered, please email it in .pdf or .tif format to clowe@aspb.org. An image title, brief, 2-3 sentence description of the image, and the name of the person credited for the image are also encouraged. If your image is selected to appear on the online cover of the journal, you will be contacted with the scheduled issue date and further information.

Thank you and congratulations on your Plant Physiology paper!

Sincerely,
Editor
Plant Physiology

=====
FIRST AUTHOR PROFILES
=====

We recognize the hard work that goes into being First Author of a Plant Physiology paper by publishing a "First Author Feature" for each First Author alongside your paper.

If the first author(s) would like to submit information for a feature on Facebook and Twitter, fill out the form by clicking on the link below:

Plant Physiology First Author Feature Form: https://forms.office.com/r/ZBCGZfhm8F
If you cannot access the form through the link above, please contact Rachel at rbelsky@aspb.org.

To upload an image of yourself to accompany your profile, please put your photo in the correct journal folder at the link below. You must label your image: Author First Name Last Name Article Title.

First Author Profile Image Dropbox: https://www.dropbox.com/scl/fo/xz029p7qjr2jml2rp2kmq/h?dl=0&rlkey=6fkxcaqmxx35ap152b2osy1fz

=====
IMPORTANT REMINDER: PEER REVIEW REPORTS
=====

If you opted to publish a peer review report along with your article during the original submission process, it will be prepared by the editorial staff and publicly posted with your manuscript, inside the zip file that contains any other supplemental material. As a reminder, the peer review report is a public record of all comments from editors and reviewers, as well as your prior responses, as you received them in the decision letters for each draft of your manuscript. If you agreed to publish this report and have changed your mind, or are not sure if you selected this option, please contact the editorial office as soon as possible before signing the license agreement from our publisher.

-- Reviewer comments:
